# Comparative genomics and metabolic model analysis of primary and secondary endosymbionts present in different species of whitefly

**DOI:** 10.1093/jisesa/ieag083

**Published:** 2026-08-08

**Authors:** Neeti Pandey, Raman Rajagopal

**Affiliations:** Department of Zoology, Kalindi College, University of Delhi, New Delhi, India; Gut Biology Lab, Department of Zoology, University of Delhi, New Delhi, India

**Keywords:** Whitefly Endosymbionts, comparative genomics, biosynthetic pathway, pathway-specific metabolic model, metabolic complementation

## Abstract

*Candidatus Portiera aleyrodidarum* (hereafter *Portiera*) is the primary endosymbiont of whiteflies (Hemiptera: Aleyrodidae), residing in specialized bacteriocytes that also co-localize diverse secondary endosymbionts. This study aimed to carry out in-depth genomic analysis of different strains of *Portiera* and secondary endosymbionts namely *Arsenophonus*, *Cardinium*, *Hamiltonella*, *Rickettsia*, and *Wolbachia* obtained from different whitefly species (data obtained from NCBI). Interestingly, hierarchical clustering of the amino acid identity data resulted in a dendrogram that resolved the different strains of *Portiera* into 3 separate genetic clusters. Pan-genome analysis of endosymbionts indicates that even closely related strains of a species, when isolated from different hosts, show unique genomic compositions due to their capability to adapt to a specific environment. COG/KEGG analysis revealed that amino acid metabolism is second most represented functional category of *Portiera* genomes whereas it is almost negligible in genomic repertoire of secondary endosymbionts except in *Hamiltonella*. Biosynthetic pathway analysis shows that threonine is the only essential amino acid for which complete set of genes is present in all strains of *Portiera* followed by leucine and tryptophan which is synthesized by all except 1 (China) and 2 strains (AD-CAI and SiSi), respectively. However, both amino acid lysine and arginine could be synthesized by only 4 strains namely AD-CAI, AF-CAI, PeMo, and TV. Further, the study established that secondary endosymbionts are capable of enriching the host’s diet with protective organic compounds, B vitamins, and cofactors which is in contrast to the role of *Portiera*. Results of pathway-specific metabolic modelling were highly congruent with KEGG pathway predictions, depicting a positive correlation between the genotype and phenotype of endosymbionts.

## Introduction

Symbiotic associations are primarily categorized into 3 types, namely commensalism, parasitism, and mutualism. Insects share an exceptional mutualistic relationship with their gut symbionts wherein both interacting prokaryote–eukaryote species flourish by benefitting each other (Dillon and Dillon 2004, Moya et al. 2008, Sanchez-Contreras and Vlisidou 2008). Insects’ symbionts are classified as either Primary (P) endosymbionts, which reside in specialized bacteriocytes within the abdomen, or Secondary (S) symbionts, which may be located in the hemolymph or surrounding tissues (Douglas 2006). All primary endosymbionts across different insect species share familiar features like obligate association with their respective host with long coevolutionary history, reduced genome, loss of genes essential for a free-living lifestyle, and confinement to specialized structures that are vertically transmitted from mother to offspring (Moya et al. 2008). P-endosymbionts help their host to thrive in diverse environmental conditions via complementing host’s diet with essential amino acids (Shigenobu et al. 2000, Pérez-Brocal et al. 2006, McCutcheon and Moran 2007) or B vitamins and cofactors (Shigenobu et al. 2000, Akman et al. 2002, van Ham et al. 2003, Wu et al. 2006) and in a few cases by providing the factors considered essential for efficient host reproduction (Foster et al. 2005). Transmission of insect symbionts can be vertical or horizontal, with both primary and secondary symbionts observed to move between hosts as shown in specialized acquisition behaviors that maintain reliable environmental transmission in insect–microbial mutualisms (Villa et al. 2023). Secondary symbionts, while more variable in distribution, also exhibit genome erosion and are host-restricted, lacking many pathways required for independent survival (Mccutcheon and Moran 2011). They may coinhabit specialized structures such as bacteriocytes alongside primary endosymbionts. In such cases, they are described as intracellular secondary symbionts, since their localization has been identified, although their classification is based on their facultative nature and transmission mode (Moya et al. 2008, Sanchez-Contreras and Vlisidou 2008).

Whiteflies (Hemiptera: Aleyrodidae) represent a group of insects with global distribution and having at least 1,556 valid species described to date. Out of these 1,556 species, at least 50 are well-known pests responsible for causing significant agricultural losses (Liu et al. 2015). Whiteflies feed on the phloem sap of several economically and commercially productive crops which are rich in carbohydrates, inorganic ions, and some nonessential amino acids, but have reduced levels of essential amino acids, B vitamins, and many cofactors that are often insufficient for insect host survival (Douglas 2006). These nutrient levels can vary depending on host plant species, plant tissue, and environmental conditions (Broussard et al. 2023). To counter their nutritionally deficient diet, almost all whiteflies harbor obligate primary endosymbionts, which are confined to specialized structures known as bacteriocytes and are typically transmitted vertically (Baumann 2005, Baumann et al. 1995). Although vertical transmission predominates in whiteflies, studies in other insects demonstrate that primary symbionts can also be horizontally acquired through host-specific behaviors that ensure reliable environmental transmission (Villa et al. 2023). *Portiera*, the obligate primary endosymbiont of whiteflies, has been reported across all species due to its co-diversification with their hosts from a common ancestor. The obligate endosymbiont *Portiera* shares a long coevolutionary history with its host species and is supposed to augment the fitness of its host by providing all the essential amino acids which are lacking in its carbohydrate-rich phloem sap diet (Thao and Baumann 2004, Jing et al. 2014). In addition to the primary endosymbionts, whiteflies are also known to carry 1 or more secondary symbionts that may coexist within bacteriocytes alongside the primary endosymbionts (Gottlieb et al. 2008, Priya et al. 2012, Pandey et al. 2013, Pandey and Rajagopal 2016) or in tissues outside bacteriocytes (Brumin et al. 2012). A few studies have reported on the role of secondary endosymbionts in metabolic complementation of the host diet with essential nutrients like B vitamins, cofactors, and other significant metabolites (Rao et al. 2015, Santos-Garcia et al. 2018) while others play essential roles in the key physiological processes of host like tolerance to temperature (Montllor et al. 2002); host reproductive behavior (McGraw and O’Neill 2004); storage & recycling of nitrogen (Gil et al. 2003) and resistance to parasites (Oliver et al. 2003).

In the past decade, next-generation sequencing techniques like sequencing by synthesis (popularly known as Illumina sequencing), 16S rDNA amplicon pyrosequencing, Ion Torrent sequencing, and sequencing by ligation (SOLid) have emerged as potent cost-effective methods for generating large datasets related to the microbial communities associated with a multitude of hosts belonging to different species (Li et al. 2025). Consequently, integration with annotation pipelines enables these datasets to serve as the basis for predicting gene functions and metabolic capacities, thereby clarifying the physiological roles of microbiomes within their hosts and environments (Passi et al. 2022). Thus, with the availability of advanced sequencing and bioinformatics tools, comparative studies of large sequenced datasets have become quite simple, rapid, and cost effective. A lot of studies have been done in the past to reveal host-specific variation in the microbiome composition of different species of whiteflies and also to highlight their role in nutritional supplementation (Rao et al. 2015, Santos-Garcia et al. 2018, Lei et al. 2023). However, the studies conducted in the past were scattered and limited to either the primary endosymbiont or 1 or 2 secondary endosymbionts of a specific whitefly species at a time. The current study intends to document the entire comparative genome analysis of *Portiera*, *Arsenophonus*, *Cardinium, Hamiltonella, Rickettsia*, and *Wolbachia* strains sequenced from different biotypes of *Bemisia tabaci* as well as other species of whiteflies, namely *Aleurodicus dispersus*, *Aleurodicus floccissimus*, *Singhiella simplex*, *Trialeurodes vaporariorum*, and *Pealius mori* (data obtained from NCBI). The rationale behind selecting whiteflies to conduct the study is their ubiquitous presence and huge economic impact despite thriving on a simple sap diet, as well as their association with different endosymbionts, which makes them prominent pests of interest in agricultural biology. Even though harboring bacteriocytes with microbial symbionts is notably costly for the hosts with respect to allocation of resources, they have nevertheless established successful evolutionary relationships with endosymbionts as these impart functional advantages to the host. The objective of this study is to establish a comparative framework for the primary endosymbiont *Portiera* and its secondary symbionts in whiteflies. Specifically, we aim to: (i) characterize genome reduction and nucleotide composition to understand evolutionary constraints, (ii) analyze codon usage bias and mutational pressure to assess the role of drift versus selection, (iii) compare phylogenomic relationships and pan-genome structures to identify conserved and flexible gene repertoires, (iv) examine biosynthetic pathways to evaluate metabolic complementation among symbionts, and (v) apply pathway-specific genome-scale metabolic modeling as a formal test of symbiont function within the host. This integrated approach clarifies how genomic variation translates into functional contributions, thereby advancing our understanding of host–microbe symbiosis and trophic interactions among symbionts.

## Materials and Methods

### Sequence Retrieval

A total of 17 whole genome sequences of *Portiera* were available in the NCBI database as of March 2025. We had assessed the quality of each genome using QUAST version 5.2 to extract assembly statistics including contig number, largest contig, total length, N50, L50, and GC%. (Gurevich et al. 2013). In addition, BUSCO v5.7.1 with the bacteria_odb10 dataset (*n* = 124) was used to evaluate gene completeness (Manni et al. 2021). Based on these metrics, only 12 high-quality genomes assemblies of *Portiera* belonging to different strains and having reference to the insect (host) from which it was isolated were selected for further pan-genome analysis (Supplementary Table S1). Here, high quality refers to assemblies with acceptable contig statistics (N50, L50, genome size, GC%) and BUSCO ­completeness scores. Assemblies with excessive fragmentation or very low completeness were excluded from further analysis. Genomic sequences of secondary endosymbionts of whiteflies—*Arsenophonus* (3), *Cardinium* (1), *Hamiltonella* (2), *Rickettsia* (1), and *Wolbachia* (2)—were also retrieved from NCBI database and their quality was similarly assessed using QUAST for assembly statistics and BUSCO for gene completeness.

### Nucleotide Composition and Codon Usage Analysis

Overall nucleotide composition (A%, G%, C%, and T%) and GC content of bacterial strains was analyzed using MEGA7 (Kumar et al. 2016). Relative Synonymous Codon Usage (RSCU) values were estimated using the CUSP program (EMBOSS version 6.6.0.0) (Rice et al. 2000). Custom Python scripts were used to assess mutational pressure (Van Rossum and Drake 2009). These scripts calculated GC3 and AT3 content, correlation coefficients (GC_r, AT_r), and codon tracking percentages for each strain (Supplementary Table S8). Genus specific averages were then derived to summarize mutational trends. Negative correlations indicated mutation driven codon bias, while positive correlations reflected base composition effects. To statistically validate these patterns, paired *t*-tests were performed to compare GC3 and AT3 values across strains (Zar 2014). Correspondence analysis (CA) was conducted to visualize codon usage variation among genera (Greenacre 1984).

### Phylogenomic Analysis

Pairwise average amino acid identity (AAI) values were computed among the genomes using MMseqs2 (Steinegger and Söding 2017). The resulting AAI matrix was converted into a distance matrix and clustered using the Neighbor-Joining (NJ) algorithm (Saitou and Nei 1987). The combined dendrogram and heatmap were visualized in Python using Seaborn to demonstrate patterns of genomic similarity and subgroup formation (Waskom 2021).

### Pan–Core Genome Analysis

The full GenBank files of genome assemblies downloaded from the NCBI served as input GenBank files (one per bacterial species) for BPGA-1.3.0 pipeline (bacterial pan-genome analysis) (Chaudhari et al. 2016). BPGA-1.3.0 pipeline processed the input file for orthologous cluster analysis. The output file obtained was used for generating gene families by USEARCH with 75% sequence similarity as a cutoff. The size of the pan-genome was calculated using the Power equation [*f*(*x*) = *a* . *x*^*b*}] using a built-in program of the BPGA-1.3.0 pipeline. In the equation, *f*(*x*) specifies total number of non-orthologous gene families within its pangenome, *x* specifies the number of bacterial strains of a particular bacterial species which are used for pan-genome analysis, *a* and *b* are fitting parameters. Additionally, the exponential equation [*f*1(*x*) = *c* . *e*^(*d . x*)] calculated the core genome data where *f*1(*x*) represents entire core gene families while *c* and *d* are fitting parameters. To minimize bias from the order in which genomes were added, 100 random permutations were incorporated during curve fitting, and 95% confidence intervals were calculated for the *b*-parameter from the asymptotic standard errors reported by BPGA to provide reliable estimates of pan-genome openness.

### Functional Analysis

Protein function was predicted via the functional analysis module of BPGA against the COG database of prokaryotic proteins (Chaudhari et al. 2016). KEGG analysis was carried out via BlastKOALA (Kanehisa et al. 2016).

### Biosynthetic Pathway Analysis

Biosynthetic pathway analysis was carried out by integrating BlastKOALA module annotations with KofamKOALA functional assignments. BlastKOALA outputs were parsed to extract module identifiers, required Kos and genome assignments, providing the structural framework for pathway analysis of essential amino acids, B vitamins as well as other cofactors present in endosymbionts of whitefly species (Kanehisa et al. 2016). Module completeness was then assessed genome-wise by verifying the presence of all required KO identifiers at each defined step of the pathway. KofamKOALA results provided quantitative evidence including adaptive thresholds, alignment scores, and *e*-values which enabled robust KO validation (Aramaki et al. 2020). The 2 datasets were merged to retain the highest scoring KO hit per genome, after which hits were classified using an adaptive threshold scheme. Hits were considered strong if they met or exceeded the KO-specific HMM threshold or fell within 10% to 20% below the cutoff (≥0.8 to 0.9 × threshold) with significant *e*-values (≤0.01) while all others were classified as missing.

### Construction and Analysis of Pathway-Specific Metabolic Models

Genome-wide KO annotations obtained from BlastKOALA and validated with KofamKOALA were parsed and grouped by genome identifiers for *Portiera*. These KO entries were mapped to curated KEGG module definitions for biosynthesis of essential amino acids. Module logic was encoded stepwise to capture enzyme requirements, complexes, and alternative routes, enabling systematic audits of pathway completeness. Pathways were classified as complete or incomplete based on the presence or absence of all required steps. For modules identified as complete, pathway-specific models were constructed in Python using COBRApy, incorporating the relevant metabolites and reactions together with exchange reactions and a demand reaction for the target compound. Flux balance analysis was then applied to quantify production capacity, providing functional validation of annotation-based predictions (Ebrahim et al. 2013).

## Results and Discussion

### Genome Statistics

This study consisted of a dataset of 21 annotated high-quality genome assemblies, which were obtained from 6 different species of endosymbionts of whiteflies: 12 genome assemblies from *Portiera*, 3 from *Arsenophonus*, 1 from *Cardinium*, 2 from *Hamiltonella*, 1 from *Rickettsia*, and 2 from *Wolbachia* (Supplementary Table S1). All these genome assemblies were assessed for general genomic features and statistics so that only high-quality genomes could be used for further downstream analysis. The genome size among different strains of *Portiera* varies from 277.770 to 411.975 kbp (Supplementary Table S1). The highly reduced genome size of *Portiera*, when compared to free-living bacteria, reflects its obligate endosymbiotic lifestyle or co-speciation with the host species. This observation has been consistent with previous studies conducted not only on *Portiera* present in different species of whiteflies (Wernegreen 2002, Baumann 2005) but also in other obligate endosymbionts like *Carsonella ruddii* in psyllids (Baumann 2005, Nakabachi et al. 2006), *Buchnera aphidicola* in aphids (Baumann et al. 1995, Douglas 1998, Baumann 2005), and *Tremblaya princeps* in mealybugs (Baumann 2005). The potential reasons for this highly reduced genome size are thought to include constraints on acquiring new genes via horizontal transfer, imposed by the obligate association of primary endosymbionts with their hosts. In addition, the intracellular lifestyle of these symbionts over evolutionary time favors the loss of nonessential genes and the retention of a minimal, stable genome (Moran 2002, Tamas et al. 2002, Gil et al. 2003, Moran and Plague 2004, Audic et al. 2005, Wernegreen 2005, Darby et al. 2007). On the contrary, in insects, facultative endosymbionts exhibit a wide range of genome sizes; however, their genome sizes are smaller when compared to free-living bacteria but larger when compared to obligate endosymbionts (Moran et al. 2005, Lamelas et al. 2008, 2011, Burke and Moran 2011). Evolutionarily, the association of whiteflies with facultative endosymbionts is more recent than their stable obligate relationship with *Portiera* (Andreason et al. 2020). According to genomic statistics (Supplementary Table S1), the genome size of the ARAD strain of *Arsenophonus*, obtained from *A. disperses*, shows extreme genome reduction (663.125 kbp) when compared to other strains like ARAF and MED-Q2 of *Arsenophonus* (3,001.875 and 1,858.967 kbp, respectively) extracted from *A. floccissimus* and *B. tabaci* Q2, respectively. The genome reduction in the ARAD strain of *Arsenophonus* is comparable to that of the primary endosymbiont *Portiera* (Supplementary Table S1). Our result analysis for ARAD strain is in agreement with a previous study conducted on this particular strain of *Arsenophonus* (Santos-Garcia et al. 2018). This highly reduced genome size of ARAD strain of *Arsenophonus* might be a step toward achieving obligate primary endosymbiosis similar to that of *Portiera* with its host species. Also, the BTQ strain of *Cardinium* endosymbiont of *B. tabaci* shows mild reduction in genome size (993.648 kbp) when compared to genome size of other facultative endosymbionts (Supplementary Table S1). The genome size of different secondary endosymbionts of whiteflies, ie *Hamiltonella* sp. lies between 1,739 kbp (MED strain) and 1,793 kbp (MEAM1 strain); *Rickettsia* sp. is about 1,214 kbp and that of *Wolbachia* sp. lies between 1,251 kbp (China 1) and 1,306 kbp (wBt-Med). As compared to the highly reduced genome of the primary symbiont *Portiera*, these facultative symbionts remain host-restricted and metabolically dependent but have experienced genome reduction over a shorter evolutionary timeframe. They retain relatively larger genomes and, unlike *Portiera*, are capable of horizontal transmission and can occupy multiple host species (Boyle et al. 1993, Huigens et al. 2004).

To complement assembly statistics, genome completeness was evaluated using BUSCO v5.7.1 with the bacteria_odb10 dataset (*n* = 124) (Supplementary Table S1). Primary symbionts (*Portiera*) consistently exhibited low BUSCO completeness, ranging from 44% to 55% across most strains (eg BT-QVLC, BT-B, PeMo, TV), with a high proportion of missing orthologs (∼40% to 42%). These results are not indicative of poor assembly or sequencing quality but rather reflect the extensive gene loss which is a characteristic feature of obligate endosymbionts, where many universal bacterial genes are genuinely absent due to long-term reductive evolution. Similar patterns have been documented in other highly reduced symbionts such as *Carsonella* and *Tremblaya*, underlining that low BUSCO scores are a biological signature of reductive evolution rather than technical deficiency (Moran and Bennett 2014). Secondary symbionts displayed substantially higher BUSCO scores which are highly consistent with their larger genomes and facultative lifestyles (Supplementary Table S1). *Hamiltonella defensa* assemblies were nearly complete (∼96% to 98%), confirming their genomic integrity and retention of conserved bacterial orthologs. *Rickettsia* (MEAM1) and *Cardinium* (BTQ) showed intermediate completeness (∼75% to 77%), reflecting moderate reduction. The genus *Arsenophonus* displayed a wide spectrum of completeness, ranging from markedly reduced genomes in ARAD (∼70%) and ARAF (∼66%) to a relatively gene-rich profile in MED-Q2 (∼88%). The ARAD strain, with its compact genome of ∼663 kbp, represents a lineage that has undergone extensive reductive evolution toward an obligate role in *Aleurodicus dispersus*. In contrast, ARAF and MED-Q2 retain much larger genomes (1.8 to 3.0 Mb); however, their BUSCO scores differ in the proportion of intact orthologs preserved. The lower BUSCO completeness observed in ARAF, despite its larger genome size, is likely a consequence of extensive pseudogenization and the proliferation of mobile elements. These features expand the overall genome length but simultaneously diminish the proportion of functional single-copy orthologs that BUSCO is able to reliably detect. These contrasting patterns highlight divergent evolutionary trajectories within *Arsenophonus* where ARAD reflects a highly reduced obligate symbiont, while ARAF and MED-Q2 remain facultative and preserve broader gene repertoires (Santos-Garcia et al. 2018). Both, *Wolbachia* genomes displayed moderate BUSCO completeness (China1 ∼82%, wBt-MED ∼78%). Their fragmented assemblies indicate structural limitations, even though they retain a relatively intact gene repertoire. Overall, the BUSCO results underscore the evolutionary divide between obligate and facultative symbionts. Thus, low scores in *Portiera* and ARAD *Arsenophonus* reflect genuine reductive evolution, whereas the high completeness of *Hamiltonella* and the moderate values observed in *Wolbachia* and *Rickettsia* confirm that secondary symbiont assemblies remain sufficiently robust for comparative analyses.

Further, nucleotide composition and GC content analysis was carried out for the CDS region of all endosymbionts considered in this study using MEGA7 (Kumar et al. 2016). Result analysis of *Portiera* genomes shows that nucleotide “A” is the most abundant with a mean value of 41.35%, followed by T (33.11%), G (15.22%), and C (10.31%) (Supplementary Table S2). Further analysis revealed that the first letter GC content (33.95%) is significantly higher than the second letter GC (29.56%) and third letter GC content (13.10%) (Supplementary Table S2). The mean AT content is calculated to be 74.46% which is significantly higher than the GC content, ie 25.54% only (Supplementary Table S2). The GC% of all secondary endosymbionts is higher when compared to *Portiera* (25.54%) and is estimated to be 38.46% in *Arsenophonus*, 37.50% in *Cardinium*, 41.18% in *Hamiltonella*, 32.53% in *Rickettsia*, and 34.42% in *Wolbachia* (Supplementary Table S2). Paired *t*-tests comparing GC3 and AT3 values across all 6 endosymbiont genera confirmed that GC3 values were significantly lower than AT3 (*t* = −6.96, *P* < 0.001). This reinforces the strong A/T bias observed in reduced symbionts, particularly *Portiera.* A very low GC content (25.54%) of *Portiera* points toward an obligate relationship with the host as observed in primary endosymbionts of other insect species too (Baumann et al. 1995, Douglas 1998, Baumann 2005, Nakabachi et al. 2006). However, the secondary endosymbionts have undergone less genome reduction with more prospects for horizontal gene transfer as well as have retained ample genes which are essential for both cellular and metabolic pathways in contrast to the primary endosymbiont *Portiera* (Bennett et al. 2024, Waterworth et al. 2020). All these factors could have culminated to the accumulation of genes with higher genomic GC content in secondary endosymbionts of whiteflies.

### Codon Usage Analysis

Proteins are composed of 20 standard amino acids which are represented by 64 codons, out of which 3 codons are stop codons. All the amino acids are encoded by multiple synonymous codons except 2 codons namely methionine and tryptophan which are encoded by unique codon. However, it has been observed that a few synonymous codons are favored over others for encoding particular amino acids. This is referred to as the codon usage bias where different synonymous codons encode same amino acid with different frequency. Codon usage bias plays vital role in determining the divergence in genome sequence as well as indicates positive, purifying or neutral selection in protein-coding genes. In protein-coding genes, synonymous mutations which are also known as silent mutations occur more often when compared to non-synonymous substitutions. Synonymous substitutions does not alter the primary structure of protein-coding genes; however, they are known to contribute significantly to the evolution of genomes (Shabalina et al. 2013, Bailey et al. 2021, Oelschlaeger 2024). In this study, codon usage bias analysis was carried out for the coding region of all endosymbionts via 3 complementary approaches. RSCU value is basically a parameter of measuring the codon usage bias (Sharp and Li 1986). A calculated RSCU value > 1 indicates a positive codon bias (RSCU value > 1.6 indicates overrepresentation of a codon or a very strong positive codon bias), whereas an RSCU value < 1 indicates a negative codon bias (RSCU value < 0.6 indicates underrepresentation of a codon) and a calculated RSCU value of “1” points toward no codon usage bias (Beelagi 2021). For instance, in *Portiera*, TTA (leucine, RSCU value = 4.35) and AGA (arginine, RSCU = 2.96) show the strongest positive codon bias whereas for other overrepresented codons RSCU values vary in a range of 1.61 to 1.96. Further, in *Portiera*, CTC (leucine, RSCU value = 0.036), CTG (leucine, RSCU value = 0.066), and TTC (phenylalanine, RSCU = 0.092) show the lowest RSCU values whereas for other underrepresented codons the RSCU values fall in a range of 0.136 to 0.558 as shown in Supplementary Table S3. Analysis of coding region of all genome assemblies depicts that the total count of 23, 9, 8, 6, 12, and 7 codons were overrepresented (RSCU value > 1.6) whereas 31, 16, 19, 13, 25, and 24 codons were underrepresented (RSCU value < 0.6) in *Portiera*, *Arsenophonus*, *Cardinium*, *Hamiltonella*, *Rickettsia*, and *Wolbachia*, respectively (Table 1). The presence of high number of over- and underrepresented codons in *Portiera* when compared to other secondary endosymbionts could possibly be due to its strict vertical transmission to next generation which leads to not only highly reduced genome size but also less stringent selection pressure. This could lead to profound genetic drift and hence considerable impact on codon usage patterns (Shields 1990, Rao et al. 2011, McCutcheon et al. 2024). Notably, the pronounced codon bias in *Portiera* is also mechanistically linked to its restricted tRNA repertoire, where the differential retention of tRNA isomers constrains codon usage patterns. This has been demonstrated in reduced bacterial symbionts, where loss of specific tRNAs forces biased codon usage (Hansen and Moran 2012).

**Table 1.** Codon usage bias and mutational pressure indicators across endosymbiont genera

Genus	GC_r	AT_r	CodonTracking%	Overrepresented codons (RSCU > 1.6)	Underrepresented codons (RSCU < 0.6)
***Portiera***	0.04	−0.05	0	23	31
***Arsenophonus***	−0.08	−0.17	1.46	9	16
***Cardinium***	−0.12	−0.2	0	8	19
***Hamiltonella***	−0.1	−0.11	11.95	6	13
***Rickettsia***	0.19	0.08	0	12	25
***Wolbachia***	0.01	−0.04	0.26	7	24

Column 1: Name of genus. Column 2: GC_r—correlation between GC3 content and codon usage indices. Column 3: AT_r—correlation between AT3 content and codon usage indices. Column 4: CodonTracking%—percentage of codons deviating from mutational bias expectations. Column 5: Overrepresented codons (RSCU > 1.6)—codons preferentially used. Column 6: Underrepresented codons (RSCU < 0.6)—codons avoided in usage.

On the contrary, secondary endosymbionts of whiteflies reside in multiple host species, exhibit less reduction in genome size and even though they are predominantly vertically transmitted (Ferrari and Vavre 2011) yet a few studies have reported that they are capable of undergoing horizontal transmission (Chiel et al. 2009). Owing to this flexible relationship with host species, likelihood of a stringent selection pressure is pronounced leading to reduced genetic drift and less divergence in codon usage patterns. Further, RSCU analysis shows that the majority of the abundantly used codons were ending in either “A” or “T” in all the endosymbionts (Supplementary Table S3) and further “AT” content of all endosymbionts was higher than that of the “GC” content and hence it is clearly seen that there was a preference for “AT” over “GC” at third codon position. This also indicates a positive correlation between nucleotide and codon composition analysis. Further, the mutation bias analysis carried out via custom Python scripts supported these findings. *Portiera* and *Cardinium* showed near zero codon tracking percentages, confirming drift dominance whereas *Arsenophonus* displayed weak correlations (GC_r −0.08, AT_r −0.17) with minimal tracking, while *Hamiltonella* exhibited modest codon tracking (∼12%), though still largely mutation biased (Table 1). *Rickettsia* showed positive GC correlation (+0.19), consistent with base composition effects, while *Wolbachia* exhibited weak signals and very low tracking (∼0.26%) (Table 1). Together, these results indicate that codon usage bias in reduced endosymbiont genomes is largely shaped by mutational pressure and genetic drift, with selection playing only a minor role. CA of codon usage patterns accounted for ∼84% of the total variance (component 0 = 67.4%, component 1 = 16.8%), based on RSCU values. The first component primarily reflected the AT versus GC bias, distinctly separating *Portiera* from secondary symbionts. The second component captured *Hamiltonella*’s unique codon usage profile, consistent with its elevated codon tracking, while the remaining genera formed a cohesive cluster indicative of balanced codon usage. These results reinforce the evolutionary divide between obligate symbionts, whose codon bias is shaped largely by drift and mutational pressure, and facultative symbionts, which retain genomic robustness and show evidence of adaptive optimization (Supplementary Fig. S1). Thus, our complementary approaches demonstrate that *Portiera*, *Cardinium*, *Rickettsia*, and *Wolbachia* display strong bias dominated by genetic drift with little evidence of translational optimization, whereas *Hamiltonella* shows partial adaptation to host tRNA pools, a pattern consistent with previous reports on whitefly symbiont genomes (Latorre et al. 2015, Marchi et al. 2018, Santos-Garcia et al. 2018, Parvathy et al. 2022).

### Phylogenomic Analysis

**Fig. 1.**
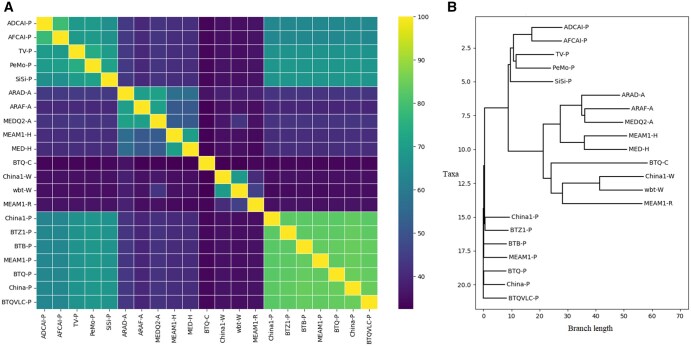
Average AAI heatmap and NJ dendrogram of Whitefly’s symbiont genomes. The heatmap (A) shows pairwise AAI values, with yellow indicating higher similarity and purple indicating lower similarity. The dendrogram (B) illustrates clustering based on the AAI distance matrix. Branch lengths on the horizontal axis represent AAI-based genetic distances, while the vertical axis lists taxa labels. Taxon labels include strain identifiers followed by a suffix denoting the symbiont group: P, *Portiera*; A, *Arsenophonus*; H, *Hamiltonella*; W, *Wolbachia*; C, *Cardinium*; R, *Rickettsia*.

Pairwise AAI values were calculated using MMseqs2 which considers reciprocal best hit orthologs through fast and sensitive protein level comparisons. For each genome pair, the mean AAI of all bidirectional best matches was used as the AAI estimate. For *Portiera*, average AAI values among the 12 observed genomes ranged from approximately 65% to 90% when self-comparisons (100% AAI) were excluded (Fig. 1). Higher AAI values (>87%) were observed among closely related strains of *Portiera* which were isolated from *B. tabaci* or between ADCAI and AFCAI strains (>83%) isolated from *Aleurodicus* sp. whereas more distantly related pairs showed values ranging from 65% to 79% (Fig. 1). Hierarchical clustering of the AAI distance matrix of *Portiera* via NJ algorithm resulted in a comprehensible dendrogram that resolved the 12 genomes into 3 distinct host-linked clusters: one comprising isolates from *B.tabaci*, a second containing the *Aleurodicus* strains (ADCAI and AFCAI), and a third cluster from *Singhiella simplex*, *Trialeurodes vaporariorum*, and *Pealius mori* (Fig. 1). This indicates that *Portiera* isolated from distant hosts shows unique genomic composition. Our findings are completely consistent with the genetic grouping reported in a previous study on *Portiera* (Lei et al. 2023). Further, AAI values among the genomes of *Arsenophonus* or *Hamiltonella* or *Wolbachia* range approximately between 69% and 72% (Fig. 1). This level of similarity is far below the conventional threshold of ∼95% for species delineation, but above the 65% boundary often used to separate genera (Mende et al. 2013). Thus, even though these genomes share a conserved evolutionary background yet they have diverged adequately to represent different species.

### Comparative Genome Analysis

**Fig. 2.**
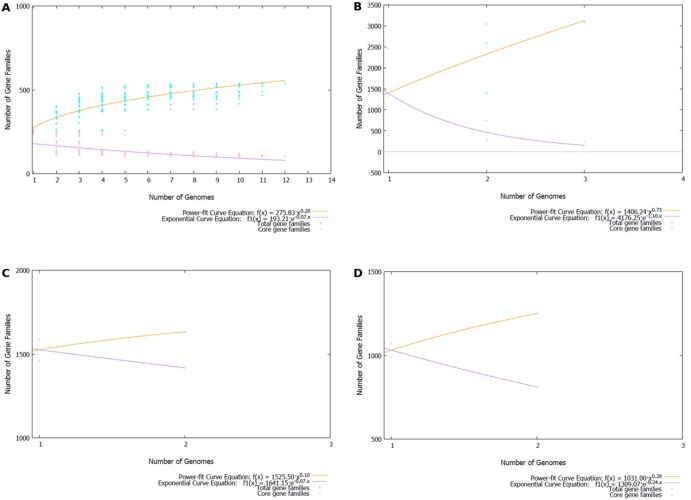
Pan–core genome analysis. Figure represents the pan and core genome curves at Usearch protein identity cut off value of 75%. Upper curve (orange curve) represents pan–genome curve whereas lower curve (blue curve) represents core–genome curve. A) Pan–core curves of *Portiera*, B) pan–core curves of *Arsenophonus*, C) pan–core curves of *Hamiltonella*, and D) pan–core curves of *Wolbachia*.

High-quality genome assemblies of endosymbionts of different species of whiteflies namely *Portiera* (12), *Arsenophonus* (3), *Hamiltonella* (2), and *Wolbachia* (2) were used separately as input data files for the BPGA-1.3.0 pipeline (Chaudhari et al. 2016). Pan–core comparative genome analysis could not be carried out for both *Cardinium* and *Rickettsia* as the genome sequence of a single strain (obtained from whitefly species) was available in the NCBI database for both these secondary endosymbionts. The protein-coding sequences of all strains of a specific endosymbiont were grouped into gene families by the USEARCH program included within the BPGA pipeline. In order to determine a fitting clustering threshold, multiple protein identity cutoffs (70%, 75%, and 80%) were assessed. A USEARCH cutoff of 75% protein identity was selected for downstream analyses since thresholds in the range of 70% to 80% are widely recommended for defining orthologous clusters in genus-level comparative genomics, particularly when genomes exhibit AAI values between 65% and 90% as seen in this study (Mende et al. 2013, Qin et al. 2014, Rouli et al. 2015, Chaudhari et al. 2016). This 75% threshold value offers a balance between retaining true orthologs and simultaneously avoiding the merging of more divergent homologs. For instance, in *Portiera*, at 70% cutoff value, BPGA pipeline identified 141 core clusters, while increasing the threshold to 75% reduced the core gene families to 102. A slight further increase in cutoff value to 80% resulted in only 52 core clusters (Supplementary Figs. S2–S4). This progressive decline in the number of core gene families in *Portiera* clearly reflects the increasing stringency of ortholog definition. Thus, using a 75% USEARCH cutoff value provided not only biologically realistic but also methodologically robust estimates for the current dataset (Fig. 2). A total of 3,039 protein-coding sequences of all *Portiera* genomes were grouped into 537 gene families among which 102 gene families (18.99% of the pan genome) represent the core gene families whereas only 143 gene families (approximately 26.62% of pan genome) and 292 gene families (54.37% of pan genome) depict the unique and accessory gene families, respectively (Supplementary Table S4). Individual genome-specific distribution of the unique, core, and accessory genes in all the strains of primary and secondary endosymbionts (analyzed via BPGA pipeline) is presented in Supplementary Table S5. The total number of gene families obtained from a specific endosymbiont was then plotted against the number of genome assemblies considered (for that particular endosymbiont) to obtain pan-genome data. Likewise, core-genome data were obtained by plotting the shared/core gene families against the number of selected genome assemblies in the current study. While generating pan–core genome data, 100 random permutations were incorporated during curve fitting to minimize bias from genome addition order. The pan–core genome data were obtained by using power equation [*f*(*x*) = *a* . *x*^*b*}] and exponential equation [*f*1(*x*) = *c* . *e*^(*d* . *x*)], respectively. In the power equation, an exponent *b* having value less than 0 specifies that the pan-genome is “closed.” Conversely, *b* value between 0 and 1 indicates that the pan-genome is still “open.” Confidence intervals (95%) were calculated for all *b* values across endosymbionts to provide reliable and comparable estimates of pan-genome openness. The pan-genome profile of *Portiera* is still open but might close soon with a *b* value of 0.281 (95% CI: 0.266 to 0.298), reflecting a narrow interval due to the relatively large number of genomes analyzed when compared to other endosymbionts (Supplementary Table S4). Further, the pan–core statistics of other secondary endosymbionts (analyzed via BPGA pipeline) have been calculated in the same manner as explained above and mentioned in Supplementary Table S4. Comparative genome analysis of *Arsenophonus* shows a very low percentage of core gene families (7.87% of pan-genome) due to extremely reduced genome size of ARAD strain of *Arsenophonus*. The total number of protein-coding genes in *Arsenophonus* strains varies from 392 to 2,465 with lowest being present in ARAD strain. The inclusion of the markedly reduced ARAD genome (∼663 kbp) disproportionately lowers the apparent core size and inflates estimates of pan-genome diversity, reflecting genuine reductive evolution rather than technical artifact. Consequently, interpretation of pan–core statistics must be made with caution in the context of pronounced genome size heterogeneity among strains. This suggests not only greater genomic diversity of the species but also its capability to adapt efficiently in different niches and potentially be the reason for open pan-genome of *Arsenophonus* (*b* value of 0.726, 95% CI: 0.679 to 0.761). Thus, it is evident that the core genome is highly conserved in a species and is engaged in performing basic cellular functions whereas the presence of a substantial percentage of flexible genes (both unique and accessory genes) might play significant role in adapting to the specific biology/life styles of host species (Grozdanov et al. 2004). Further, approximately 86.83% and 64.96% of the pan-genome of *Hamiltonella* and *Wolbachia*, respectively, represent core gene families which indicate a conserved genomic pool with low diversity and thus indicate toward gene fixation. For *Hamiltonella*, the *b* value was 0.0982 (95% CI: −0.100 to 0.296), consistent with a nearly closed pan-genome but with a wide interval which reflects the limited number of genomes available, underscoring the need for expanded sequencing to refine estimates. *Wolbachia*, meanwhile, exhibited a moderately open pan-genome with *b* value of 0.2779 (95% CI: 0.110 to 0.446), again reflecting moderate precision constrained by small sample size. Thus, the final results might vary slightly between studies considering the protocol followed, sample size, origin of genomes, software used, and the extrapolation performed (Vernikos 2020). Hence, this outlines the necessity to sequence and consider larger amounts of genomic data obtained from all strains of endosymbionts present in different species of whiteflies to obtain a clearer picture of conserved and dispensable genes.

### COG/KEGG Analysis

**Fig. 3.**
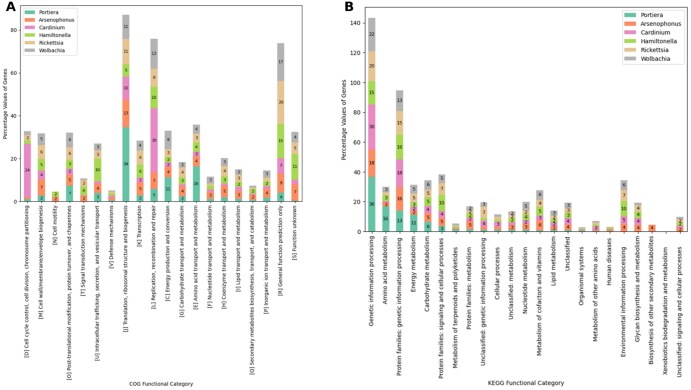
Distribution of functional COG/KEGG categories. Figure represents the percentage distribution of (A) COG functional categories (B) KEGG functional categories in genes of primary and secondary endosymbionts of whiteflies.

Protein-coding genes of *Portiera* and other secondary endosymbionts were classified to establish their clusters of orthologous groups (COGs) identities. The distribution of the major COG categories for all endosymbionts is depicted in Fig. 3. The 2 most prominent COG functional categories in *Portiera* genomes are Translation, ribosomal structure, and biogenesis [J] (34.59%) and Amino acid transport and metabolism [E] (16.32%). Further, detailed analysis of *Portiera* COGs subcategories show that 2 other categories occupying fair share of genes are Replication, recombination and repair [L] (5.80%) and Energy production and conversion [C] (11.25%). This indicates that reduced genome of primary endosymbiont *Portiera* primarily focuses on amino acid metabolism besides maintaining central information machinery. As expected, all the secondary endosymbionts namely *Arsenophonus* (4.72%), *Cardinium* (2.07%), *Hamiltonella* (4.45%), *Rickettsia* (3.93%), and *Wolbachia* (4.34%) have moderately low share of genes involved in Amino acid transport and metabolism [E] when compared to primary endosymbiont *Portiera*. Additionally, it can be said that percentage of genes in different COG categories is more evenly distributed in all secondary endosymbionts when compared to *Portiera*. COG analysis focuses on the functional categorization of genes based on their evolutionary relationship. The above observations imply that genes which are involved in maintaining basic and essential cellular functions as well as few metabolic functions are retained in all the endosymbionts whereas those involved in complex processes like signaling, cell motility, chromatin structure, and RNA processing are either highly reduced or entirely lost in all endosymbionts. Additionally, KEGG pathway analysis determines the metabolic repertoire of the organism. As shown in figure, the highest percentage of genes is involved in Genetic information processing in *Portiera* as well as in other endosymbionts (Fig. 3). The second most represented KEGG functional category is amino acid metabolism in *Portiera* with around 16.82% of genes involved in it. However, this percentage is extremely low in all other secondary endosymbionts with highest being 3.99% in *Hamiltonella* (Fig. 3). Further, the percentage of genes involved in metabolism of cofactors and vitamins is almost negligible in *Portiera* (0.93%) whereas a significant proportion of genes of secondary endosymbionts specifically *Arsenophonus* (6.58%), *Cardinium* (3.24%), *Hamiltonella* (5.70%), *Rickettsia* (4.89%), and *Wolbachia* (6.43%) are involved in this particular functional category. The percentage share of all other KEGG functional categories for all other endosymbionts is shown in Fig. 3. Based on above COG/KEGG results, it is understood that the highly reduced genome of obligate endosymbiont *Portiera* retains several genes which are responsible for amino acid metabolism yet lacks those involved in cofactors/vitamins synthesis and hence clearly emphasizes on the complementation between *Portiera* and its secondary endosymbionts where both are dependent upon each other to fulfill each other’s as well as host species cellular and metabolic needs.

### Biosynthetic Pathway Analysis

To mitigate annotation errors in reduced symbiont genomes, we manually inspected of key biosynthetic genes and further combined automated BlastKOALA annotations with KofamKOALA threshold-based validation. Stepwise diagnostic tables (per KO score, threshold, and *e*-value) were generated for each module which enabled identification of borderline hits and potential pseudogenes. Modules with BlastKOALA–KofamKOALA disagreements were manually examined to confirm whether discrepancies reflected true gene loss, threshold effects, or annotation mismatches. Insects require at least 10 essential amino acids from the diet which include phenylalanine, valine, threonine, tryptophan, methionine, leucine, isoleucine, lysine, histidine, and arginine (Singh and Brown 1957). We performed the biosynthetic pathway analysis of different genes retained in all strains of *Portiera* in order to deduce whether it was capable of synthesizing the essential amino acid and other vitamins/cofactors necessary for its host species (Table 2). Our results showed that threonine is the only essential amino acid synthesized by all strains of *Portiera* considered in this study. Further genes for biosynthesis of tryptophan are present in all the strains of *Portiera* except in 2 strains namely AD-CAI and SiSi. Similarly, genes for biosynthesis of leucine are present in all strains except one, ie China. Interestingly, only 4 strains of *Portiera* have the complete set of genes for the synthesis of amino acid lysine (AD-CAI, AF-CAI, PeMo, and TV). Further, none of the *Portiera* strains obtained from *B. tabaci* biotypes is capable of synthesizing amino acid arginine; however, all the other 5 strains (AD-CAI, AF-CAI, SiSi, PeMo, and TV) obtained from other species of whiteflies have the complete set of genes for the synthesis of arginine. Further, all the strains lack 1 or 2 genes for the synthesis of 5 other essential amino acids namely phenylalanine, valine, methionine, isoleucine, and histidine (Table 2). Interestingly, all the strains have complete set of genes for Shikimate pathway. The end product of this pathway is chorismate which serves as the precursor for the synthesis of essential amino acids like phenylalanine and tryptophan. Thus, it is possible that all the strains have lost a few genes during the course of evolution and hence all the species of whitefly observed in this study were incapable of synthesizing phenylalanine. Thus, BlastKOALA and KofamKOALA analyses are in agreement across all 10 essential amino acid biosynthetic modules with the only discrepancy was observed in lipoic acid biosynthesis while BlastKOALA annotated this pathway as complete in ADCAI and TV, whereas KofamKOALA scanning classified it as incomplete based on threshold criteria. When quantified across all modules and genomes, both annotation systems agreed in 98.33% of cases, with only 1.66% showing disagreement. These disagreements were restricted to lipoic acid biosynthesis, highlighting threshold driven differences rather than widespread annotation mismatches (Table 2). In conclusion, the biosynthetic machinery of *Portiera* strains regarding production of vitamins/cofactors as well as Beta-Carotene is rather insufficient. The inability of the *Portiera* strains to synthesize all 10 essential amino acids is partially contrary to the notion that the major role of the primary endosymbiont is to enrich the nutritionally deficient diet of the host insect. Further, results suggest that even though the primary endosymbiont can still potentially synthesize a few essential amino acids yet it entirely failed to complement the host species diet with B vitamins and cofactors. Our results are in agreement with many studies conducted in the past that have proven the inability of the primary endosymbionts of insects to enrich host’s suboptimal diet with all essential nutrients (Tamames et al. 2007, McCutcheon and Moran 2010, Sloan et al. 2014). Thus, it can be proposed that there is a possibility that lost function of primary endosymbiont is compensated by some other secondary endosymbiont which cohabits along with primary endosymbiont since whiteflies lack the genes to synthesize these essential components of diet.

**Table 2.** Biosynthetic profile for 10 essential amino acids and lipoic acid in 12 strains of *P. aleyrodidarum*

Genome_ID	Amino_Acid_Module	KOALA_Complete	Kofam_Complete	Agreement
**ADCAI**	Arginine	Complete	Complete	Agree
**AFCAI**	Arginine	Complete	Complete	Agree
**BTB**	Arginine	Incomplete	Incomplete	Agree
**BTQ**	Arginine	Incomplete	Incomplete	Agree
**BTQVLC**	Arginine	Incomplete	Incomplete	Agree
**BTZ1**	Arginine	Incomplete	Incomplete	Agree
**China**	Arginine	Incomplete	Incomplete	Agree
**China1**	Arginine	Incomplete	Incomplete	Agree
**MEAM1**	Arginine	Incomplete	Incomplete	Agree
**PeMo**	Arginine	Complete	Complete	Agree
**SiSi**	Arginine	Complete	Complete	Agree
**TV**	Arginine	Complete	Complete	Agree
**ADCAI**	Leucine	Complete	Complete	Agree
**AFCAI**	Leucine	Complete	Complete	Agree
**BTB**	Leucine	Complete	Complete	Agree
**BTQ**	Leucine	Complete	Complete	Agree
**BTQVLC**	Leucine	Complete	Complete	Agree
**BTZ1**	Leucine	Complete	Complete	Agree
**China**	Leucine	Incomplete	Incomplete	Agree
**China1**	Leucine	Complete	Complete	Agree
**MEAM1**	Leucine	Complete	Complete	Agree
**PeMo**	Leucine	Complete	Complete	Agree
**SiSi**	Leucine	Complete	Complete	Agree
**TV**	Leucine	Complete	Complete	Agree
**ADCAI**	Lipoic acid	Complete	Incomplete	Disagree
**AFCAI**	Lipoic acid	Incomplete	Incomplete	Agree
**BTB**	Lipoic acid	Incomplete	Incomplete	Agree
**BTQ**	Lipoic acid	Incomplete	Incomplete	Agree
**BTQVLC**	Lipoic acid	Incomplete	Incomplete	Agree
**BTZ1**	Lipoic acid	Incomplete	Incomplete	Agree
**China**	Lipoic acid	Incomplete	Incomplete	Agree
**China1**	Lipoic acid	Incomplete	Incomplete	Agree
**MEAM1**	Lipoic acid	Incomplete	Incomplete	Agree
**PeMo**	Lipoic acid	Incomplete	Incomplete	Agree
**SiSi**	Lipoic acid	Incomplete	Incomplete	Agree
**TV**	Lipoic acid	Complete	Incomplete	Disagree
**ADCAI**	Lysine	Complete	Complete	Agree
**AFCAI**	Lysine	Complete	Complete	Agree
**BTB**	Lysine	Incomplete	Incomplete	Agree
**BTQ**	Lysine	Incomplete	Incomplete	Agree
**BTQVLC**	Lysine	Incomplete	Incomplete	Agree
**BTZ1**	Lysine	Incomplete	Incomplete	Agree
**China**	Lysine	Incomplete	Incomplete	Agree
**China1**	Lysine	Incomplete	Incomplete	Agree
**MEAM1**	Lysine	Incomplete	Incomplete	Agree
**PeMo**	Lysine	Complete	Complete	Agree
**SiSi**	Lysine	Incomplete	Incomplete	Agree
**TV**	Lysine	Complete	Complete	Agree
**ADCAI**	Threonine	Complete	Complete	Agree
**AFCAI**	Threonine	Complete	Complete	Agree
**BTB**	Threonine	Complete	Complete	Agree
**BTQ**	Threonine	Complete	Complete	Agree
**BTQVLC**	Threonine	Complete	Complete	Agree
**BTZ1**	Threonine	Complete	Complete	Agree
**China**	Threonine	Complete	Complete	Agree
**China1**	Threonine	Complete	Complete	Agree
**MEAM1**	Threonine	Complete	Complete	Agree
**PeMo**	Threonine	Complete	Complete	Agree
**SiSi**	Threonine	Complete	Complete	Agree
**TV**	Threonine	Complete	Complete	Agree
**ADCAI**	Tryptophan	Incomplete	Incomplete	Agree
**AFCAI**	Tryptophan	Complete	Complete	Agree
**BTB**	Tryptophan	Complete	Complete	Agree
**BTQ**	Tryptophan	Complete	Complete	Agree
**BTQVLC**	Tryptophan	Complete	Complete	Agree
**BTZ1**	Tryptophan	Complete	Complete	Agree
**China**	Tryptophan	Complete	Complete	Agree
**China1**	Tryptophan	Complete	Complete	Agree
**MEAM1**	Tryptophan	Complete	Complete	Agree
**PeMo**	Tryptophan	Complete	Complete	Agree
**SiSi**	Tryptophan	Incomplete	Incomplete	Agree
**TV**	Tryptophan	Complete	Complete	Agree
**ADCAI**	Histidine	Incomplete	Incomplete	Agree
**AFCAI**	Histidine	Incomplete	Incomplete	Agree
**BTB**	Histidine	Incomplete	Incomplete	Agree
**BTQ**	Histidine	Incomplete	Incomplete	Agree
**BTQVLC**	Histidine	Incomplete	Incomplete	Agree
**BTZ1**	Histidine	Incomplete	Incomplete	Agree
**China**	Histidine	Incomplete	Incomplete	Agree
**China1**	Histidine	Incomplete	Incomplete	Agree
**MEAM1**	Histidine	Incomplete	Incomplete	Agree
**PeMo**	Histidine	Incomplete	Incomplete	Agree
**SiSi**	Histidine	Incomplete	Incomplete	Agree
**TV**	Histidine	Incomplete	Incomplete	Agree
**ADCAI**	Methionine	Incomplete	Incomplete	Agree
**AFCAI**	Methionine	Incomplete	Incomplete	Agree
**BTB**	Methionine	Incomplete	Incomplete	Agree
**BTQ**	Methionine	Incomplete	Incomplete	Agree
**BTQVLC**	Methionine	Incomplete	Incomplete	Agree
**BTZ1**	Methionine	Incomplete	Incomplete	Agree
**China**	Methionine	Incomplete	Incomplete	Agree
**China1**	Methionine	Incomplete	Incomplete	Agree
**MEAM1**	Methionine	Incomplete	Incomplete	Agree
**PeMo**	Methionine	Incomplete	Incomplete	Agree
**SiSi**	Methionine	Incomplete	Incomplete	Agree
**TV**	Methionine	Incomplete	Incomplete	Agree
**ADCAI**	Valine	Incomplete	Incomplete	Agree
**AFCAI**	Valine	Incomplete	Incomplete	Agree
**BTB**	Valine	Incomplete	Incomplete	Agree
**BTQ**	Valine	Incomplete	Incomplete	Agree
**BTQVLC**	Valine	Incomplete	Incomplete	Agree
**BTZ1**	Valine	Incomplete	Incomplete	Agree
**China**	Valine	Incomplete	Incomplete	Agree
**China1**	Valine	Incomplete	Incomplete	Agree
**MEAM1**	Valine	Incomplete	Incomplete	Agree
**PeMo**	Valine	Incomplete	Incomplete	Agree
**SiSi**	Valine	Incomplete	Incomplete	Agree
**TV**	Valine	Incomplete	Incomplete	Agree
**ADCAI**	Phenyalanine	Incomplete	Incomplete	Agree
**AFCAI**	Phenyalanine	Incomplete	Incomplete	Agree
**BTB**	Phenyalanine	Incomplete	Incomplete	Agree
**BTQ**	Phenyalanine	Incomplete	Incomplete	Agree
**BTQVLC**	Phenyalanine	Incomplete	Incomplete	Agree
**BTZ1**	Phenyalanine	Incomplete	Incomplete	Agree
**China**	Phenyalanine	Incomplete	Incomplete	Agree
**China1**	Phenyalanine	Incomplete	Incomplete	Agree
**MEAM1**	Phenyalanine	Incomplete	Incomplete	Agree
**PeMo**	Phenyalanine	Incomplete	Incomplete	Agree
**SiSi**	Phenyalanine	Incomplete	Incomplete	Agree
**TV**	Phenyalanine	Incomplete	Incomplete	Agree
**ADCAI**	Isoleucine	Incomplete	Incomplete	Agree
**AFCAI**	Isoleucine	Incomplete	Incomplete	Agree
**BTB**	Isoleucine	Incomplete	Incomplete	Agree
**BTQ**	Isoleucine	Incomplete	Incomplete	Agree
**BTQVLC**	Isoleucine	Incomplete	Incomplete	Agree
**BTZ1**	Isoleucine	Incomplete	Incomplete	Agree
**China**	Isoleucine	Incomplete	Incomplete	Agree
**China1**	Isoleucine	Incomplete	Incomplete	Agree
**MEAM1**	Isoleucine	Incomplete	Incomplete	Agree
**PeMo**	Isoleucine	Incomplete	Incomplete	Agree
**SiSi**	Isoleucine	Incomplete	Incomplete	Agree
**TV**	Isoleucine	Incomplete	Incomplete	Agree

Whiteflies host diverse communities of maternally inherited secondary (facultative) endosymbionts in addition to the obligate primary endosymbiont. Studies conducted in the past have indicated that 7 genera of secondary endosymbiotic bacteria are predominantly associated with whiteflies which include *Arsenophonus*, *Cardinium*, *Hamiltonella*, *Rickettsia*, *Wolbachia*, *Hemipteriphilus*, and *Fritchea* (Zchori-Fein and Brown 2002, Banks et al. 2003, Weeks et al. 2003, Everett et al. 2005, Gottlieb et al. 2006, Priya et al. 2012, Bing et al. 2013, Pandey and Rajagopal 2016). To test out the nutritional role of secondary endosymbionts in whiteflies, we performed the biosynthetic pathway analysis of different genes retained in all strains of the above-mentioned secondary endosymbionts associated with different species of whiteflies. Merged BlastKOALA–KofamKOALA analysis with manual inspection revealed broad agreement across most genomes for essential amino acid and cofactor biosynthetic modules. Annotation analysis via BLAST method revealed that only *Hamiltonella defensa* strains obtained from MED and MEAM biotypes of *B. tabaci* were capable of synthesizing essential amino acid threonine. However, Kofam analysis confirmed completeness of biosynthetic pathway of threonine in MEAM, but in MED it reported the pathway to be incomplete, leading to disagreement between the 2 annotation systems. Further, proline biosynthesis in ARAF (*Arsenophonus* endosymbiont of *A. floccissimus*/ARAF) and the shikimate pathway in MEAM (*Candidatus Hamiltonella defensa* of *B. tabaci/*MEAM) were consistently complete with agreement. None of the other strains of secondary endosymbiont was capable of contributing any essential or nonessential amino acid to the diet of their host species (Supplementary Table S6). Further, results depict that glutathione biosynthesis which acts as powerful antioxidant and hence detoxifies potentially toxic substances that might hamper the growth and development of host species (Allen and Sohal 1986) is confirmed in ARAF (*Arsenophonus* endosymbiont of *Aleurodicus floccissimus*/ARAF), MEDQ2 (*Arsenophonus* endosymbiont of *Bemisia tabaci* Q2/MED-Q2) and MEAM (*Candidatus Hamiltonella defensa* of *Bemisia tabaci*/MEAM1), but MED (*Candidatus Hamiltonella defensa* MED of *Bemisia tabaci*/MED), wBt-MED (*Wolbachia* endosymbiont of *Bemisia tabaci* strain wBt-MED) and China1 (*Wolbachia* endosymbiont of *Bemisia tabaci*/China1) show disagreement, reflecting threshold-based downgrades despite BLAST-based KOALA completeness. Insects usually cannot produce micronutrients themselves and hence are dependent upon microbial symbionts as their phloem sap diet is rich in carbohydrates but lacks essential amino acids, cofactors and B vitamins. Cofactors and B vitamins assist in catalyzing the vital metabolic reactions and cellular processes in insects (Serrato-Salas and Gendrin 2023, Kaltenpoth et al. 2025). The Table depicts that all the secondary endosymbionts are capable of synthesizing different cofactors and B vitamins essential for the survival, growth and development of host species (Supplementary Table S6). For instance, lipoic acid which is an organosulfur compound was synthesized by all secondary endosymbionts with consistency (except MED strain of *Candidatus Hamiltonella defensa* of *B. tabaci*) acting as a cofactor for several key enzymes that are involved in the process of energy metabolism as well as cellular protection of the insect host (Kaczmarek et al. 2024). Further, results depict that microbial symbionts have retained the capability to synthesize key B vitamins like thiamine, riboflavin, pyridoxal, folate, etc., and hence were able to nutritionally contribute to host’s diet. For instance, biotin, pyridoxal phosphate, tetrahydrofolate, and thiamine biosynthesis were complete in ARAF strain with agreement. MEAM (*Candidatus Hamiltonella defensa* of *Bemisia tabaci*/MEAM1) exhibited complete NAD, ubiquinone, coenzyme A, pimeloyl-ACP, lipoic acid, and PreQ1 biosynthesis, all confirmed by both annotation systems. In contrast, MED (*Candidatus Hamiltonella defensa* MED of *Bemisia tabaci*/MED) showed systematic disagreements across NAD, ubiquinone, coenzyme A, pimeloyl-ACP, lipoic acid, PreQ1, and riboflavin, where BLAST-based KOALA annotated completeness but threshold-based Kofam inspection rejected borderline hits. MEDQ2 was largely concordant but displayed disagreement for riboflavin biosynthesis. Overall, BlastKOALA and KofamKOALA classifications agreed in 70.27% of cases with 29.72% showing disagreement (Supplementary Table S6). These disagreements were largely concentrated in MED and MEDQ2 genomes, particularly affecting cofactor and vitamin pathways, underscoring the importance of threshold-based validation and manual curation to distinguish genuine pathway loss from borderline annotations. Studies conducted in the past have also shown the significance of B vitamins as coenzymes, imparting enhanced immunity to host, increasing both lifespan and fecundity of host as well as increased resistance to diseases (Serrato-Salas and Gendrin 2023). Thus, based on above observations, secondary endosymbionts at large are incapable of complementing host’s diet with amino acids; however, they are also equally significant as they could potentially enrich the nutritionally poor diet of host with B vitamins for optimal growth and development as well as with cofactors which might help in adapting to different environmental conditions (Rao et al. 2015, Opatovsky et al. 2018, Santos-Garcia et al. 2018, Wang et al. 2020, Selvaraj et al. 2021). The list of all genes involved in key metabolic pathways of both primary and secondary endosymbionts along with BlastKOALA/KofamKOALA annotation data is provided in Supplementary Table S7. In essence, each symbiont of the host may specialize in specific metabolic pathways, allowing them to jointly meet the host’s requirements. Thus, COG/KEGG and biosynthetic analysis provide insights into the dynamics of host–endosymbiont relationship and its crucial role in whitefly survival as well as adaptation.

### In Silico Pathway-Specific Metabolic Model Reconstruction and Determining Prototrophy/Auxotrophy Statistics

Pathway-specific metabolic models were constructed only for those essential amino acid biosynthesis modules (threonine, tryptophan, arginine, leucine, lysine) that were annotated as complete in at least some *Portiera* genomes by both annotation systems (Table 2). These sub-models defined KEGG accurate reactions and metabolites for each pathway, together with exchange and demand reactions, and were analyzed using flux balance analysis to validate production capacity (Supplementary Table S8). Genomes in which flux production was observed can thus be considered prototrophic for the synthesis of that specific amino acid, whereas those lacking flux are auxotrophic and dependent on host or co-symbiont complementation. Primarily, our pathway-specific metabolic model results are in strong agreement with the BlastKOALA/KofamKOALA analysis (Tables 2 and 3). These highly consistent results suggest a strong confirmation between the genomic potential of the organism and its biological or metabolic capabilities (phenotype) under controlled simulated physiological conditions produced using computational tools. Result analysis revealed that threonine biosynthesis was universally complete across all genomes, while tryptophan was incomplete in ADCAI and SiSi due to loss of key enzymes (Table 3). Lysine biosynthesis was retained only in ADCAI, AFCAI, PeMo, and TV, with other strains missing 1 or more key enzymes of the biosynthetic pathway (Table 3). Arginine biosynthesis was complete in 5 genomes (ADCAI, AFCAI, PeMo, SiSi, and TV) but disrupted in other genomes due to the absence of argininosuccinate lyase. Leucine biosynthesis was broadly intact, except in the China strain, which lacked acetohydroxyacid isomeroreductase (Table 3). The universal retention of threonine suggests strong selective pressure to preserve this pathway, while variable losses in tryptophan, lysine, and arginine reflect strain-specific metabolic dependencies. These findings highlight uneven retention of essential amino acid pathways in *Portiera*, which is consistent with the progressive genomic reduction and metabolic complementation with host as well as co-symbionts (Rao et al. 2015). Further, flux balance analysis of the phenylalanine biosynthesis pathway showed that all *Portiera* genomes lacked 2 key enzymes, namely, chorismate mutase and aromatic transaminase, resulting in no flux toward phenylalanine under minimal-medium conditions (Supplementary Table S9). This confirmed that all *Portiera* genomes are auxotrophic for phenylalanine when considered in isolation. However, when exchange reactions were introduced to simulate host derived supplementation of missing intermediates (prephenate or phenylpyruvate), flux to phenylalanine was restored to nonzero values indicating metabolic complementation (Table 4). Thus, all *Portiera* genomes therefore become prototrophic for phenylalanine only when host metabolites are available. This computational result mirrors experimental evidence of a recent study that observed that all strains of *Portiera* have all the genes which are essential for the synthesis of phenylalanine except the terminal GOT2 (Glutamate Oxaloacetate Transaminase 2) which is required in the final step of its production. However, this gene is amply expressed in insect’s bacteriocytes which utilize phenylpyruvate as the precursor and glutamate as the amino donor (Lv et al. 2025). Together, these findings highlight the critical role of host–symbiont cooperation in sustaining amino acid biosynthesis despite symbiont genome reduction.

**Table 3.** Pathway-specific metabolic models for threonine, tryptophan, lysine, arginine, and leucine biosynthesis across *Portiera* genomes. Each entry indicates whether the pathway is complete or incomplete, together with the flux balance analysis outcome (flux value of 1,000.0 for complete pathways; no flux for incomplete pathways). When incomplete, the specific missing enzyme(s) are listed. Enzyme names are provided under each pathway heading to show the steps required for biosynthetic functionality

Genome	**Threonine** (Aspartokinase, Aspartate_semialdehyde_dehydrogenase, Homoserine_dehydrogenase, Homoserine_kinase, Threonine_synthase)	**Tryptophan** (Anthranilate_synthase, Phosphoribosyltransferase, Tryptophan_synthase)	**Lysine** (Aspartate_kinase, Aspartate_semialdehyde_dehydrogenase, HTPA_synthase, HTPA_reductase, Succinyl transferase, Aminotransferase, Desuccinylase, Epimerase, Decarboxylase)	**Arginine** (Ornithine_carbamoyltransferase, Argininosuccinate_synthase, Argininosuccinate_lyase)	**Leucine** (Acetolactate_synthase, Acetohydroxyacid_isomeroreductase, Dihydroxyacid_dehydratase)
**ADCAI**	Complete, flux 1,000.0 (all enzymes present)	Incomplete, no flux (missing anthranilate synthase)	Complete, flux 1,000.0 (all enzymes present)	Complete, flux 1,000.0 (all enzymes present)	Complete, flux 1,000.0 (all enzymes present)
**AFCAI**	Complete, flux 1,000.0 (all enzymes present)	Complete, flux 1,000.0 (all enzymes present)	Complete, flux 1,000.0 (all enzymes present)	Complete, flux 1,000.0 (all enzymes present)	Complete, flux 1,000.0 (all enzymes present)
**BTB**	Complete, flux 1,000.0 (all enzymes present)	Complete, flux 1,000.0 (all enzymes present)	Incomplete, no flux (missing succinyltransferase, epimerase, decarboxylase)	Incomplete, no flux (missing argininosuccinate lyase)	Complete, flux 1,000.0 (all enzymes present)
**BTQ**	Complete, flux 1,000.0 (all enzymes present)	Complete, flux 1,000.0 (all enzymes present)	Incomplete, no flux (same missing steps as BTB)	Incomplete, no flux (missing argininosuccinate lyase)	Complete, flux 1,000.0 (all enzymes present)
**BTQVLC**	Complete, flux 1,000.0 (all enzymes present)	Complete, flux 1,000.0 (all enzymes present)	Incomplete, no flux (same missing steps as BTB)	Incomplete, no flux (missing argininosuccinate lyase)	Complete, flux 1,000.0 (all enzymes present)
**BTZ1**	Complete, flux 1,000.0 (all enzymes present)	Complete, flux 1,000.0 (all enzymes present)	Incomplete, no flux (same missing steps as BTB)	Incomplete, no flux (missing argininosuccinate lyase)	Complete, flux 1,000.0 (all enzymes present)
**China**	Complete, flux 1,000.0 (all enzymes present)	Complete, flux 1,000.0 (all enzymes present)	Incomplete, no flux (same missing steps as BTB)	Incomplete, no flux (missing argininosuccinate lyase)	Incomplete, no flux (missing isomeroreductase)
**China1**	Complete, flux 1,000.0 (all enzymes present)	Complete, flux 1,000.0 (all enzymes present)	Incomplete, no flux (same missing steps as BTB)	Incomplete, no flux (missing argininosuccinate lyase)	Complete, flux 1,000.0 (all enzymes present)
**MEAM1**	Complete, flux 1,000.0 (all enzymes present)	Complete, flux 1,000.0 (all enzymes present)	Incomplete, no flux (same missing steps as BTB)	Incomplete, no flux (missing argininosuccinate lyase)	Complete, flux 1,000.0 (all enzymes present)
**PeMo**	Complete, flux 1,000.0 (all enzymes present)	Complete, flux 1,000.0 (all enzymes present)	Complete, flux 1,000.0 (all enzymes present)	Complete, flux 1,000.0 (all enzymes present)	Complete, flux 1,000.0 (all enzymes present)
**SiSi**	Complete, flux 1,000.0 (all enzymes present)	Incomplete, no flux (missing phosphoribosyltransferase)	Incomplete, no flux (missing epimerase, decarboxylase)	Complete, flux 1,000.0 (all enzymes present)	Complete, flux 1,000.0 (all enzymes present)
**TV**	Complete, flux 1,000.0 (all enzymes present)	Complete, flux 1,000.0 (all enzymes present)	Complete, flux 1,000.0 (all enzymes present)	Complete, flux 1,000.0 (all enzymes present)	Complete, flux 1,000.0 (all enzymes present)

**Table 4.** Genome-wise analysis of phenylalanine biosynthesis in *Portiera*. Presence or absence of key enzymes (*chorismate mutase*, *prephenate dehydratase*, *aromatic transaminase*) was determined from KOALA/KofamScan annotations. “Module_complete” indicates whether all 3 enzymes are present. “Flux_to_Phenylalanine” represents flux balance analysis results under minimal medium conditions, showing no production in any genome. “Flux_with_Complementation” shows flux values when host-derived intermediates (prephenate or phenylpyruvate) were supplied via exchange reactions, restoring phenylalanine production to 1,000.0 in all genomes

Genome	Chorismate_mutase	Prephenate_dehydratase	Aromatic_transaminase	Module_complete	Flux_to_Phenylalanine	Flux_with_Complementation
**ADCAI**	FALSE	TRUE	FALSE	FALSE	0	1,000
**AFCAI**	FALSE	TRUE	FALSE	FALSE	0	1,000
**BTB**	FALSE	TRUE	FALSE	FALSE	0	1,000
**BTQ**	FALSE	TRUE	FALSE	FALSE	0	1,000
**BTQVLC**	FALSE	TRUE	FALSE	FALSE	0	1,000
**BTZ1**	FALSE	TRUE	FALSE	FALSE	0	1,000
**China**	FALSE	TRUE	FALSE	FALSE	0	1,000
**China1**	FALSE	TRUE	FALSE	FALSE	0	1,000
**MEAM1**	FALSE	TRUE	FALSE	FALSE	0	1,000
**PeMo**	FALSE	TRUE	FALSE	FALSE	0	1,000
**SiSi**	FALSE	TRUE	FALSE	FALSE	0	1,000
**TV**	FALSE	TRUE	FALSE	FALSE	0	1,000

## Conclusion

Thus, to conclude, in the current study, we have presented the pan-genome analysis and metabolic pathway analysis of primary and secondary endosymbionts associated with different species of whiteflies. Firstly, the study has led to a better understanding of these obligate and facultative endosymbionts and provides a valuable framework in understanding that even closely related strains of a bacterial species display wide variation in genomic composition in accordance with the host environment. However, there is a need for sequencing other strains of these endosymbionts across diverse species of whiteflies to achieve a more comprehensible genome pool. Secondly, biosynthetic pathways in *Portiera* and other secondary endosymbionts highlight the interdependency between co-symbionts. Being endosymbionts, they rely on insect host for safe accommodation and better nutrition but simultaneously enrich nutritionally not only the host’s diet nutritionally but also could possibly supplement each other’s diets as well. Thirdly, *Portiera* encodes metabolic genes which are primarily involved in the biosynthesis of essential amino acids but lacks genes for the substantial production of significant metabolites like B vitamins and cofactors whereas secondary endosymbionts encodes considerable number of genes which participate in synthesis of protective organic compounds, essential B vitamins, and regulatory cofactors. It is highly possible that secondary endosymbionts have taken over the role of primary endosymbiont for a few metabolic functions in some of the whitefly species. This has led to the emergence of mutualistic relationship between the primary and secondary endosymbionts of the whitefly species.

## Supplementary Material

ieag083_Supplementary_Data

## Data Availability

The datasets used in the current study are available at NCBI.
