# Supplementary material for: Comparative genomics and metabolic model analysis of primary and secondary endosymbionts present in different species of whitefly
Source: J Insect Sci. 2026 Aug 8;26(4):ieag083. doi: 10.1093/jisesa/ieag083 (PMC13452057; doi:10.1093/jisesa/ieag083)
Supplement: Supplementary file 1 [file ieag083_supplementary_data.zip › Supplementary Material Legends.pdf]

Supplementary Material

Supplementary Figure 1: Correspondence Analysis (CA) of codon usage bias in endosymbiont genomes. Ordination plot showing the distribution of six endosymbiont genera (*Portiera, Arsenophonus, Cardinium, Hamiltonella, Rickettsia,* and *Wolbachia*) based on Relative Synonymous Codon Usage (RSCU) values. The first axis (Component 0, 67.4% variance explained) reflects the AT versus GC bias, clearly separating *Portiera* from secondary symbionts. The second axis (Component 1, 16.8% variance explained) highlights *Hamiltonella's* distinct codon usage profile, consistent with its elevated codon tracking. The remaining genera cluster together, indicative of balanced codon usage patterns. Each point represents the centroid of codon usage for a given genome, with proximity reflecting similarity in codon bias.

Supplementary Figure 2: Pan-Core genome analysis. Figure represents the pan and core genome curves at Usearch protein identity cut off value of 70%. Upper curve (orange curve) represent pan-genome curve whereas lower curve (blue curve) represents core-genome curve. Panel (A) depicts Pan-Core curves of *Portiera*; Panel (B) depicts Pan-Core curves of *Arsenophonus*; Panel (C) depicts Pan-Core curves of *Hamiltonella*; Panel (D) depicts Pan-Core curves of *Wolbachia*.

Supplementary Figure 3: Pan-Core genome analysis. Figure represents the pan and core genome curves at Usearch protein identity cut off value of 75%. Upper curve (orange curve) represent pan-genome curve whereas lower curve (blue curve) represents core-genome curve. Panel (A) depicts Pan-Core curves of *Portiera*; Panel (B) depicts Pan-Core curves of *Arsenophonus*; Panel (C) depicts Pan-Core curves of *Hamiltonella*; Panel (D) depicts Pan-Core curves of *Wolbachia*.

Supplementary Figure 4: Pan-Core genome analysis. Figure represents the pan and core genome curves at Usearch protein identity cut off value of 80%. Upper curve (orange curve) represent pan-genome curve whereas lower curve (blue curve) represents core-genome curve. Panel (A) depicts Pan-Core curves of *Portiera*; Panel (B) depicts Pan-Core curves of *Arsenophonus*; Panel (C) depicts Pan-Core curves of *Hamiltonella*; Panel (D) depicts Pan-Core curves of *Wolbachia*.

Supplementary Table S1: Details of bacterial strains used, general genomic features and assembly statistics of primary and secondary endosymbionts obtained from different species of whiteflies.

Supplementary Table S2: Nucleotide composition analysis of six endosymbionts obtained from whiteflies.

Supplementary Table S3: Table depicts RSCU codon analysis values in primary and secondary endosymbionts of whiteflies.

Supplementary Table S4: Table depicts species specific Pan-Core genome statistics of endosymbionts obtained from whiteflies.

Supplementary Table S5: Table depicts genome-wise distribution of core, unique and accessory genes in primary and secondary endosymbionts of whiteflies.

Supplementary Table S6: Table depicts biosynthetic profile for amino acids, co-factors and B-Vitamins of secondary endosymbionts of whiteflies.

Supplementary Table S7: List of genes involved in synthesis of metabolites in primary and secondary endosymbionts of whiteflies

Supplementary Table S8: Details of scripts used for constructing pathway specific metabolic models of essential amino acids.

Supplementary Table S9: Table depicts exchange bounds for metabolites in the phenylalanine biosynthesis model under minimal and host-supplemented media.
