# Supplementary material for: Comparative genomics and metabolic model analysis of primary and secondary endosymbionts present in different species of whitefly
Source: J Insect Sci. 2026 Aug 8;26(4):ieag083. doi: 10.1093/jisesa/ieag083 (PMC13452057; doi:10.1093/jisesa/ieag083)
Supplement: Supplementary file 1 [file ieag083_supplementary_data.zip › Supplementary Table S1.docx]

| **S.No** | **Scientific Name of Organism** | **Source of isolation** | **Strain Name** | **Assembly ID** | **Assembly level** | **Total number of contigs** | **Largest contig** | **Total length** | **N50** | **L50** | **GC (%)** | **BUSCO Complete (%)** | **Fragmented (%)** | **Missing (%)** |
| --- | --- | --- | --- | --- | --- | --- | --- | --- | --- | --- | --- | --- | --- | --- |
| 1 | *Candidatus* Portiera aleyrodidarum BT-QVLC | *Bemisia tabaci* Q | BT-QVLC | ASM30003v1 | Complete Genome | 1 | 357472 | 357472 | 357472 | 1 | 26.12 | 46 | 12.1 | 41.9 |
| 2 | *Candidatus* Portiera aleyrodidarum | *Bemisia tabaci* (Biotype Q) | china | CAAS_CanPor1.0 | Chromosome | 1 | 375688 | 375688 | 375688 | 1 | 26.04 | 44.3 | 12.9 | 42.8 |
| 3 | *Candidatus* Portiera aleyrodidarum MED (*Bemisia tabaci*) | *Bemisia tabaci* | BT-Q | ASM82785v1 | Complete Genome | 1 | 357461 | 357461 | 357461 | 1 | 26.12 | 46 | 12.1 | 41.9 |
| 4 | *Candidatus* Portiera aleyrodidarum MED (*Bemisia tabaci*) | *Bemisia tabaci* | MEAM1 | ASM228587v1 | Complete Genome | 1 | 352068 | 352068 | 352068 | 1 | 26.22 | 46 | 12.1 | 41.9 |
| 5 | *Candidatus* Portiera aleyrodidarum | *Bemisia tabaci* China 1 | China_1 | ASM284999v1 | Complete Genome | 1 | 359359 | 359359 | 359359 | 1 | 26.1 | 46 | 12.1 | 41.9 |
| 6 | *Candidatus* Portiera aleyrodidarum | *Bemisia tabaci* Asia II 3. | BT-Z1 | ASM284997v1 | Complete Genome | 1 | 354523 | 354523 | 354523 | 1 | 26.1 | 46.8 | 11.3 | 41.9 |
| 7 | *Candidatus* Portiera aleyrodidarum BT-B-HRs | *Bemisia tabaci* (biotype B) | BT-B | ASM29268v1 | Complete Genome | 1 | 358242 | 358242 | 358242 | 1 | 26.17 | 46 | 12.1 | 41.9 |
| 8 | *Candidatus* Portiera aleyrodidarum | *Aleurodicus dispersus* ADAW-CAI | AD-CAI | AD-CAI.1 | Complete Genome | 1 | 290195 | 290195 | 290195 | 1 | 23.94 | 54 | 6.5 | 39.5 |
| 9 | *Candidatus* Portiera aleyrodidarum | *Aleurodicus floccissimus* AFAW-CAI | AF-CAI | AF-CAI.1 | Complete Genome | 1 | 280822 | 280822 | 280822 | 1 | 24.69 | 54.8 | 5.6 | 39.6 |
| 10 | *Candidatus* Portiera aleyrodidarum | *Singhiella simplex* | SiSi | SISI | Contig | 9 | 177293 | 411975 | 170981 | 2 | 26.18 | 51.6 | 5.6 | 42.8 |
| 11 | *Candidatus* Portiera aleyrodidarum TV | *Trialeurodes vaporariorum* | TV | ASM34974v1 | Complete Genome | 1 | 280663 | 280663 | 280663 | 1 | 24.69 | 53.2 | 5.6 | 41.2 |
| 12 | *Candidatus* Portiera aleyrodidarum | *Pealius mori* | PeMo | PEMO_1 | Complete Genome | 1 | 277700 | 277700 | 277700 | 1 | 24.1 | 54.8 | 4.8 | 40.4 |
| 13 | *Arsenophonus* endosymbiont of *Aleurodicus dispersus* | *Aleurodicus dispersus* | ARAD | ARADv1 | Complete | 1 | 663125 | 663125 | 663125 | 1 | 32.19 | 70.2 | 1.6 | 28.2 |
| 14 | *Arsenophonus* endosymbiont of *Aleurodicus floccissimus* | *Aleurodicus floccissimus* | ARAF | ARAF1v1 | Contig | 11 | 1306380 | 3001875 | 510728 | 2 | 36.95 | 66.1 | 0.8 | 33.1 |
| 15 | *Arsenophonus* endosymbiont of *Bemisia tabaci* Q2 | *Bemisia tabaci* Q2 | MED-Q2 | ARBTMED-Q2 | Contig | 324 | 67861 | 1858967 | 10733 | 44 | 36.87 | 87.9 | 0.8 | 11.3 |
| 16 | *Cardinium* endosymbiont of *Bemisia tabaci* | *Bemisia tabaci* (biotype Q) | BTQ | CAAS_CanCar1.0 | Scaffold | 3 | 993648 | 996809 | 993648 | 1 | 36.15 | 75 | 4 | 21 |
| 17 | *Candidatus* Hamiltonella defensa MED of *Bemisia tabaci* | *Bemisia tabaci* | MED | ASM25834v2 | Contig | 1 | 1739504 | 1739504 | 1739504 | 1 | 40.3 | 96.8 | 1.6 | 1.6 |
| 18 | *Candidatus* Hamiltonella defensa of *Bemisia tabaci* | *Bemisia tabaci* | MEAM1 | ASM228585v1 | Complete | 72 | 214721 | 1793760 | 102662 | 7 | 40.45 | 97.6 | 1.6 | 0.8 |
| 19 | *Rickettsia sp.* MEAM1 *Bemisia tabaci* | *Bemisia tabaci* | MEAM1 | ASM228590v1 | Complete | 201 | 39533 | 1214693 | 8566 | 39 | 32.2 | 77.4 | 2.4 | 20.2 |
| 20 | *Wolbachia* endosymbiont of *Bemisia tabaci* | *Bemisia tabaci* China 1 | China 1 | ASM399958v1 | Chromosome | 297 | 29205 | 1251074 | 6274 | 55 | 33.96 | 82.3 | 4.8 | 12.9 |
| 21 | *Wolbachia* endosymbiont of *Bemisia tabaci* strain wBt-MED draft genome | *Bemisia tabaci* | wBt-MED | WBTv1 | Contig | 1 | 1306495 | 1306495 | 1306495 | 1 | 33.9 | 78.2 | 8.1 | 13.7 |
