# Supplementary material for: Comparative genomics and metabolic model analysis of primary and secondary endosymbionts present in different species of whitefly
Source: J Insect Sci. 2026 Aug 8;26(4):ieag083. doi: 10.1093/jisesa/ieag083 (PMC13452057; doi:10.1093/jisesa/ieag083)
Supplement: Supplementary file 1 [file ieag083_supplementary_data.zip › Supplementary Table S2.docx]

| Organism | A% | G% | C% | T% | AT% | GC% | First letter GC%  content | Second letter GC %  content | Third letter GC %  content |
| --- | --- | --- | --- | --- | --- | --- | --- | --- | --- |
| *Portiera sp.* | 41.35 | 15.22 | 10.31 | 33.11 | 74.46 | 25.54 | 33.95 | 29.56 | 13.10 |
| *Arsenophonus sp.* | 31.52 | 20.86 | 17.60 | 30.01 | 61.54 | 38.46 | 49.21 | 36.13 | 30.05 |
| *Cardinium sp.* | 32.73 | 19.71 | 17.79 | 29.77 | 62.5 | 37.50 | 47.20 | 35.54 | 29.74 |
| *Hamiltonella sp.* | 30.36 | 21.79 | 19.39 | 28.46 | 58.82 | 41.18 | 49.95 | 36.63 | 36.97 |
| *Ricketessia sp.* | 36.24 | 18.37 | 14.16 | 31.23 | 67.47 | 32.53 | 42.83 | 32.35 | 22.40 |
| *Wolbachia sp.* | 35.87 | 19.99 | 14.43 | 29.70 | 65.58 | 34.42 | 43.89 | 33.20 | 26.18 |
