# Supplementary material for: Comparative genomics and metabolic model analysis of primary and secondary endosymbionts present in different species of whitefly
Source: J Insect Sci. 2026 Aug 8;26(4):ieag083. doi: 10.1093/jisesa/ieag083 (PMC13452057; doi:10.1093/jisesa/ieag083)
Supplement: Supplementary file 1 [file ieag083_supplementary_data.zip › Supplementary Table S3.docx]

| Codon | AA | RSCU of *Portiera* | RSCU of *Arsenophonus* | RSCU of *Cardinium* | RSCU of *Hamiltonella* | RSCU of *Ricketessia* | RSCU of *Wolbachia* |
| --- | --- | --- | --- | --- | --- | --- | --- |
| GCA | A | 1.784 | 1.264 | 1.312 | 1.02 | 1.532 | 1.904 |
| GCC | A | 0.18 | 0.672 | 0.876 | 1.02 | 0.372 | 0.268 |
| GCG | A | 0.244 | 0.676 | 0.392 | 0.856 | 0.252 | 0.328 |
| GCT | A | 1.792 | 1.384 | 1.42 | 1.104 | 1.844 | 1.504 |
| TGC | C | 0.178 | 0.626 | 0.624 | 0.704 | 0.592 | 0.742 |
| TGT | C | 1.822 | 1.374 | 1.376 | 1.296 | 1.408 | 1.258 |
| GAC | D | 0.194 | 0.308 | 0.398 | 0.486 | 0.276 | 0.394 |
| GAT | D | 1.806 | 1.692 | 1.602 | 1.514 | 1.724 | 1.606 |
| GAA | E | 1.79 | 1.51 | 1.512 | 1.474 | 1.424 | 1.412 |
| GAG | E | 0.21 | 0.49 | 0.488 | 0.526 | 0.576 | 0.588 |
| TTC | F | 0.092 | 0.354 | 0.286 | 0.356 | 0.342 | 0.368 |
| TTT | F | 1.908 | 1.646 | 1.714 | 1.644 | 1.658 | 1.632 |
| GGA | G | 1.508 | 0.756 | 1.008 | 0.884 | 1 | 1.416 |
| GGC | G | 0.288 | 0.912 | 0.816 | 1.04 | 0.692 | 0.596 |
| GGG | G | 0.38 | 0.448 | 0.576 | 0.832 | 0.484 | 0.42 |
| GGT | G | 1.82 | 1.88 | 1.596 | 1.244 | 1.824 | 1.568 |
| CAC | H | 0.258 | 0.452 | 0.394 | 0.5 | 0.314 | 0.6 |
| CAT | H | 1.742 | 1.548 | 1.606 | 1.5 | 1.686 | 1.4 |
| ATA | I | 1.617 | 0.627 | 1.068 | 0.618 | 1.248 | 1.356 |
| ATC | I | 0.144 | 0.621 | 0.504 | 0.819 | 0.354 | 0.387 |
| ATT | I | 1.239 | 1.752 | 1.428 | 1.563 | 1.398 | 1.257 |
| AAA | K | 1.77 | 1.608 | 1.492 | 1.652 | 1.54 | 1.416 |
| AAG | K | 0.23 | 0.392 | 0.508 | 0.348 | 0.46 | 0.584 |
| CTA | L | 0.36 | 0.726 | 0.96 | 0.36 | 0.846 | 0.876 |
| CTC | L | 0.036 | 0.288 | 0.312 | 0.564 | 0.138 | 0.336 |
| CTG | L | 0.066 | 0.606 | 0.45 | 0.93 | 0.264 | 0.468 |
| CTT | L | 0.558 | 0.876 | 1.026 | 0.84 | 1.218 | 1.206 |
| TTA | L | 4.35 | 2.49 | 2.01 | 2.118 | 2.946 | 2.1 |
| TTG | L | 0.624 | 1.014 | 1.236 | 1.182 | 0.6 | 1.02 |
| ATG | M | 1 | 1 | 1 | 1 | 1 | 1 |
| AAC | N | 0.314 | 0.436 | 0.522 | 0.568 | 0.366 | 0.498 |
| AAT | N | 1.686 | 1.564 | 1.478 | 1.432 | 1.634 | 1.502 |
| CCA | P | 1.66 | 1.432 | 1.396 | 0.872 | 1.372 | 1.812 |
| CCC | P | 0.184 | 0.472 | 0.66 | 0.84 | 0.196 | 0.252 |
| CCG | P | 0.228 | 0.728 | 0.296 | 0.608 | 0.604 | 0.368 |
| CCT | P | 1.928 | 1.368 | 1.644 | 1.684 | 1.828 | 1.564 |
| CAA | Q | 1.814 | 1.366 | 1.472 | 1.298 | 1.474 | 1.404 |
| CAG | Q | 0.186 | 0.634 | 0.528 | 0.702 | 0.526 | 0.596 |
| AGA | R | 2.964 | 0.84 | 1.746 | 0.96 | 2.7 | 2.844 |
| AGG | R | 0.486 | 0.294 | 0.822 | 0.462 | 0.774 | 1.122 |
| CGA | R | 0.378 | 0.852 | 0.66 | 0.918 | 0.534 | 0.438 |
| CGC | R | 0.192 | 1.056 | 0.636 | 1.032 | 0.378 | 0.438 |
| CGG | R | 0.27 | 0.576 | 0.564 | 0.576 | 0.18 | 0.144 |
| CGT | R | 1.71 | 2.388 | 1.572 | 2.052 | 1.434 | 1.014 |
| AGC | S | 0.204 | 0.876 | 0.852 | 0.894 | 0.978 | 0.9 |
| AGT | S | 1.596 | 1.506 | 1.38 | 1.044 | 1.428 | 1.566 |
| TCA | S | 1.902 | 1.398 | 0.96 | 1.218 | 1.398 | 1.428 |
| TCC | S | 0.156 | 0.426 | 0.654 | 0.582 | 0.354 | 0.312 |
| TCG | S | 0.24 | 0.528 | 0.324 | 0.546 | 0.36 | 0.288 |
| TCT | S | 1.902 | 1.266 | 1.83 | 1.71 | 1.476 | 1.506 |
| ACA | T | 1.968 | 1.12 | 1.212 | 1.148 | 1.2 | 1.56 |
| ACC | T | 0.224 | 1.004 | 1.192 | 1.108 | 0.532 | 0.416 |
| ACG | T | 0.26 | 0.576 | 0.484 | 0.704 | 0.336 | 0.356 |
| ACT | T | 1.548 | 1.3 | 1.112 | 1.04 | 1.932 | 1.664 |
| GTA | V | 1.896 | 0.996 | 1.756 | 0.724 | 1.7 | 1.464 |
| GTC | V | 0.136 | 0.548 | 0.472 | 0.904 | 0.28 | 0.28 |
| GTG | V | 0.284 | 0.68 | 0.556 | 1.184 | 0.448 | 0.692 |
| GTT | V | 1.684 | 1.772 | 1.212 | 1.188 | 1.572 | 1.56 |
| TGG | W | 1 | 1 | 1 | 1 | 1 | 1 |
| TAC | Y | 0.142 | 0.364 | 0.456 | 0.476 | 0.296 | 0.538 |
| TAT | Y | 1.858 | 1.636 | 1.544 | 1.524 | 1.704 | 1.462 |
