# Supplementary material for: Comparative genomics and metabolic model analysis of primary and secondary endosymbionts present in different species of whitefly
Source: J Insect Sci. 2026 Aug 8;26(4):ieag083. doi: 10.1093/jisesa/ieag083 (PMC13452057; doi:10.1093/jisesa/ieag083)
Supplement: Supplementary file 1 [file ieag083_supplementary_data.zip › Supplementary Table S4.docx]

| Endosymbiont | No. of genomes considered | Total no. of protein coding genes | Orthologous  clusters (Pan  genome size) | Core  Clusters  (% of Pan-genome) | Unique clusters (% of Pan-genome) | Accessory clusters (% of Pan-genome) | b-parameter [with 95% Confidence interval (CI) ] | Pan-genome status |
| --- | --- | --- | --- | --- | --- | --- | --- | --- |
| *Portiera aleyrodidarum* | 12 | 3039 | 537 | 102  (18.99% of the pan genome) | 143  (26.62% of pan genome) | 292  (54.37% of pan genome) | 0.281806 [95% CI: 0.266–0.298] | The Pan genome is still open but may be closed soon. |
| *Arsenophonus sp.* | 3 | 4184 | 3097 | 244  (7.87% of the pan genome) | 2254  (72.78% of the pan genome) | 599  (19.34  % of the pan genome) | 0.726456  [95% CI: 0.679 to 0.761] | Open |
| *Hamiltonella sp.* | 2 | 3051 | 1633 | 1418  (86.83 % of the pan genome) | 215  (13.16 % of the pan genome) | 0  (0 % of the pan genome) | 0.0982426 [95% CI: –0.100 to 0.296] | Almost closed |
| *Wolbachia sp.* | 2 | 2062 | 1250 | 812  (64.96 % of the pan genome) | 438  (35.04 % of the pan genome) | 0  (0 % of the pan genome) | 0.277884 [95% CI: 0.110–0.446] | The Pan genome is still open but may be closed soon. |
