# Supplementary material for: Comparative genomics and metabolic model analysis of primary and secondary endosymbionts present in different species of whitefly
Source: J Insect Sci. 2026 Aug 8;26(4):ieag083. doi: 10.1093/jisesa/ieag083 (PMC13452057; doi:10.1093/jisesa/ieag083)
Supplement: Supplementary file 1 [file ieag083_supplementary_data.zip › Supplementary Table S5.docx]

| Genome no. | Organism name | CDS Count | No. of Core genes | No. of Accessory genes | No. of Unique genes | % Core genes | % Accessory genes | % Unique genes |
| --- | --- | --- | --- | --- | --- | --- | --- | --- |
| 1 | Candidatus_Portiera_aleyrodidarum_AD_CAI | 272 | 102 | 100 | 70 | 37.5 | 36.765 | 25.735 |
| 2 | Candidatus_Portiera_aleyrodidarum_AF_CAI | 262 | 102 | 160 | 0 | 38.931 | 61.069 | 0 |
| 3 | Candidatus_Portiera_aleyrodidarum_BT_B_HRs | 250 | 102 | 148 | 0 | 40.8 | 59.2 | 0 |
| 4 | Candidatus_Portiera_aleyrodidarum_MED_BT_Q_Bemisia_tabaci_ | 250 | 102 | 147 | 1 | 40.8 | 58.8 | 0.4 |
| 5 | Candidatus_Portiera_aleyrodidarum_BT_QVLC | 251 | 102 | 148 | 1 | 40.637 | 58.964 | 0.398 |
| 6 | Candidatus_Portiera_aleyrodidarum_BT_Z1 | 247 | 102 | 145 | 0 | 41.296 | 58.704 | 0 |
| 7 | Candidatus_Portiera_aleyrodidarum_China1 | 238 | 102 | 134 | 2 | 42.857 | 56.303 | 0.84 |
| 8 | Candidatus_Portiera_aleyrodidarum_China | 247 | 102 | 143 | 2 | 41.296 | 57.895 | 0.81 |
| 9 | Candidatus_Portiera_aleyrodidarum_MED_MEAM1_Bemisia_tabaci_ | 246 | 102 | 143 | 1 | 41.463 | 58.13 | 0.407 |
| 10 | Candidatus_Portiera_aleyrodidarum_PeMo | 260 | 102 | 142 | 16 | 39.231 | 54.615 | 6.154 |
| 11 | Candidatus_Portiera_aleyrodidarum_SiSi | 258 | 102 | 107 | 49 | 39.535 | 41.473 | 18.992 |
| 12 | Candidatus_Portiera_aleyrodidarum_TV | 258 | 102 | 155 | 1 | 39.535 | 60.078 | 0.388 |
| 13 | ARAD_Arsenophonus_endosymbiont_of_Aleurodicus_dispersus | 392 | 244 | 96 | 52 | 62.245 | 24.49 | 13.265 |
| 14 | ARAF_Arsenophonus_endosymbiont_of_Aleurodicus_floccissimus | 2465 | 244 | 534 | 1687 | 9.899 | 21.663 | 68.438 |
| 15 | MED_Arsenophonus_endosymbiont_of_Bemisia_tabaci_Q2 | 1327 | 244 | 568 | 515 | 18.387 | 42.803 | 38.809 |
| 16 | MEAM1_Candidatus_Hamiltonella_defensa_Bemisia_tabaci_ | 1461 | 1418 | 0 | 43 | 97.057 | 0 | 2.943 |
| 17 | MED_Candidatus_Hamiltonella_defensa_MED_Bemisia_tabaci_ | 1590 | 1418 | 0 | 172 | 89.182 | 0 | 10.818 |
| 18 | china1_Wolbachia_endosymbiont_of_Bemisia_tabaci | 991 | 812 | 0 | 179 | 81.937 | 0 | 18.063 |
| 19 | WBT1_Wolbachia_endosymbiont_of_Bemisia_tabaci | 1071 | 812 | 0 | 259 | 75.817 | 0 | 24.183 |
