# Supplementary material for: Comparative genomics and metabolic model analysis of primary and secondary endosymbionts present in different species of whitefly
Source: J Insect Sci. 2026 Aug 8;26(4):ieag083. doi: 10.1093/jisesa/ieag083 (PMC13452057; doi:10.1093/jisesa/ieag083)
Supplement: Supplementary file 1 [file ieag083_supplementary_data.zip › Supplementary Table S6.docx]

| **Genome_ID** | **Name of the organism** | **Amino_Acid_Module/ B-Vitamin_Module/Co-factors_Module** | **KOALA_Complete** | **Kofam_Complete** | **Agreement** |
| --- | --- | --- | --- | --- | --- |
| ARAD | *Arsenophonus* endosymbiont of *Aleurodicus dispersus*/ARAD | Lipoic acid biosynthesis, plants and bacteria, octanoyl-ACP => dihydrolipoyl-E2/H | Complete | Complete | Agree |
| ARAF | *Arsenophonus* endosymbiont of *Aleurodicus floccissimus*/ARAF | Biotin biosynthesis, pimeloyl-ACP/CoA => biotin | Complete | Complete | Agree |
| ARAF | *Arsenophonus* endosymbiont of *Aleurodicus floccissimus*/ARAF | Pyridoxal phosphate biosynthesis, erythrose-4P => pyridoxal-P | Complete | Complete | Agree |
| ARAF | *Arsenophonus* endosymbiont of *Aleurodicus floccissimus*/ARAF | Tetrahydrofolate biosynthesis, GTP => THF | Complete | Complete | Agree |
| ARAF | *Arsenophonus* endosymbiont of *Aleurodicus floccissimus*/ARAF | Thiamine biosynthesis, prokaryotes, AIR (+ DXP/tyrosine) => TMP/TPP | Complete | Complete | Agree |
| ARAF | *Arsenophonus* endosymbiont of *Aleurodicus floccissimus*/ARAF | Lipoic acid biosynthesis, plants and bacteria, octanoyl-ACP => dihydrolipoyl-E2/H | Complete | Complete | Agree |
| ARAF | *Arsenophonus* endosymbiont of *Aleurodicus floccissimus*/ARAF | Proline biosynthesis, glutamate => proline | Complete | Complete | Agree |
| ARAF | *Arsenophonus* endosymbiont of *Aleurodicus floccissimus*/ARAF | Glutathione biosynthesis, glutamate => glutathione | Complete | Complete | Agree |
| BTQ | *Cardinium* endosymbiont of *Bemisia tabaci* (biotype Q)/BTQ | Lipoic acid biosynthesis, plants and bacteria, octanoyl-ACP => dihydrolipoyl-E2/H | Complete | Complete | Agree |
| BTQ | *Cardinium* endosymbiont of *Bemisia tabaci* (biotype Q)/BTQ | Pyridoxal phosphate biosynthesis, R5P + glyceraldehyde-3P + glutamine => pyridoxal-P) | Complete | Complete | Agree |
| MEAM | Candidatus Hamiltonella defensa of *Bemisia tabaci*/MEAM1 | NAD biosynthesis, aspartate => quinolinate => NAD | Complete | Complete | Agree |
| MEAM | Candidatus Hamiltonella defensa of *Bemisia tabaci*/MEAM1 | Ubiquinone biosynthesis, prokaryotes, chorismate (+ polyprenyl-PP) => ubiquinol | Complete | Complete | Agree |
| MEAM | Candidatus Hamiltonella defensa of *Bemisia* *tabaci*/MEAM1 | Coenzyme A biosynthesis, pantothenate => CoA | Complete | Complete | Agree |
| MEAM | Candidatus Hamiltonella defensa of *Bemisia tabaci*/MEAM1 | Pimeloyl-ACP biosynthesis, BioC-BioH pathway, malonyl-ACP => pimeloyl-ACP | Complete | Complete | Agree |
| MEAM | Candidatus Hamiltonella defensa of *Bemisia tabaci*/MEAM1 | Lipoic acid biosynthesis, plants and bacteria, octanoyl-ACP => dihydrolipoyl-E2/H | Complete | Complete | Agree |
| MEAM | Candidatus Hamiltonella defensa of *Bemisia tabaci*/MEAM1 | PreQ1 biosynthesis, GTP => 7-Aminomethyl-7-deazaguanine | Complete | Complete | Agree |
| MEAM | Candidatus Hamiltonella defensa of *Bemisia tabaci*/MEAM1 | Threonine biosynthesis, aspartate => homoserine => threonine | Complete | Complete | Agree |
| MEAM | Candidatus Hamiltonella defensa of *Bemisia tabaci*/MEAM1 | Shikimate pathway, phosphoenolpyruvate + erythrose-4P => chorismate | Complete | Complete | Agree |
| MEAM | Candidatus Hamiltonella defensa of *Bemisia tabaci*/MEAM1 | Glutathione biosynthesis, glutamate => glutathione | Complete | Complete | Agree |
| MED | Candidatus Hamiltonella defensa MED of *Bemisia tabaci*/MED | NAD biosynthesis, aspartate => quinolinate => NAD | Complete | Incomplete | Disagree |
| MED | Candidatus Hamiltonella defensa MED of *Bemisia tabaci*/MED | Ubiquinone biosynthesis, prokaryotes, chorismate (+ polyprenyl-PP) => ubiquinol | Complete | Incomplete | Disagree |
| MED | Candidatus Hamiltonella defensa MED of *Bemisia tabaci*/MED | Coenzyme A biosynthesis, pantothenate => CoA | Complete | Incomplete | Disagree |
| MED | Candidatus Hamiltonella defensa MED of *Bemisia tabaci*/MED | Pimeloyl-ACP biosynthesis, BioC-BioH pathway, malonyl-ACP => pimeloyl-ACP | Complete | Incomplete | Disagree |
| MED | Candidatus Hamiltonella defensa MED of *Bemisia tabaci*/MED | Lipoic acid biosynthesis, plants and bacteria, octanoyl-ACP => dihydrolipoyl-E2/H | Complete | Incomplete | Disagree |
| MED | Candidatus Hamiltonella defensa MED of *Bemisia tabaci*/MED | PreQ1 biosynthesis, GTP => 7-Aminomethyl-7-deazaguanine | Complete | Incomplete | Disagree |
| MED | Candidatus Hamiltonella defensa MED of *Bemisia tabaci*/MED | Threonine biosynthesis, aspartate => homoserine => threonine | Complete | Incomplete | Disagree |
| MED | Candidatus Hamiltonella defensa MED of *Bemisia tabaci*/MED | Glutathione biosynthesis, glutamate => glutathione | Complete | Incomplete | Disagree |
| MEDQ2 | *Arsenophonus* endosymbiont of *Bemisia tabaci* Q2/MED-Q2 | Coenzyme A biosynthesis, pantothenate => CoA | Complete | Complete | Agree |
| MEDQ2 | *Arsenophonus* endosymbiont of *Bemisia tabaci* Q2/MED-Q2 | Riboflavin biosynthesis, plants and bacteria, GTP => riboflavin/FMN/FAD | Complete | Incomplete | Disagree |
| MEDQ2 | *Arsenophonus* endosymbiont of *Bemisia tabaci* Q2/MED-Q2 | Lipoic acid biosynthesis, plants and bacteria, octanoyl-ACP => dihydrolipoyl-E2/H | Complete | Complete | Agree |
| MEDQ2 | *Arsenophonus* endosymbiont of *Bemisia tabaci* Q2/MED-Q2 | Glutathione biosynthesis, glutamate => glutathione | Complete | Complete | Agree |
| MEAM1 | *Rickettsia* sp. MEAM1 (*Bemisia tabaci*)/MEAM1 | Lipoic acid biosynthesis, plants and bacteria, octanoyl-ACP => dihydrolipoyl-E2/H | Complete | Complete | Agree |
| MEAM1 | *Rickettsia* sp. MEAM1 (*Bemisia tabaci)*/MEAM1 | PreQ1 biosynthesis, GTP => 7-Aminomethyl-7-deazaguanine | Complete | Complete | Agree |
| wBt-MED | *Wolbachia* endosymbiont of *Bemisia tabaci* strain wBt-MED | Lipoic acid biosynthesis, plants and bacteria, octanoyl-ACP => dihydrolipoyl-E2/H | Complete | Complete | Agree |
| wBt-MED | *Wolbachia* endosymbiont of *Bemisia tabaci* strain wBt-MED | Glutathione biosynthesis, glutamate => glutathione | Complete | Incomplete | Disagree |
| China1 | *Wolbachia* endosymbiont of *Bemisia tabaci*/China1 | Lipoic acid biosynthesis, plants and bacteria, octanoyl-ACP => dihydrolipoyl-E2/H | Complete | Complete | Agree |
| China1 | *Wolbachia* endosymbiont of *Bemisia tabaci*/China1 | Glutathione biosynthesis, glutamate => glutathione | Complete | Incomplete | Disagree |
