# Supplementary material for: Comparative genomics and metabolic model analysis of primary and secondary endosymbionts present in different species of whitefly
Source: J Insect Sci. 2026 Aug 8;26(4):ieag083. doi: 10.1093/jisesa/ieag083 (PMC13452057; doi:10.1093/jisesa/ieag083)
Supplement: Supplementary file 1 [file ieag083_supplementary_data.zip › Supplementary Table S7.pdf]

Supplementary Table S7: List of genes involved in synthesis of metabolites in primary and secondary endosymbionts of whiteflies

Genome_ID	Module	Complete	Stepwise
ADCAI	Threonine (M00018)	True
['True(K00928[score=468.5,thr=353.77,e=5.7e-141,strength=Strong])',
'True(K00133[score=339.1,thr=189.07,e=1e-101,strength=Strong])',
'True(K00003[score=498.5,thr=273.33,e=6.3e-150,strength=Strong])',
'True(K02204[score=278.3,thr=133.1,e=2.1e-83,strength=Strong])',
'True(K01733[score=323.1,thr=265.67,e=7.6e-97,strength=Strong])']
ADCAI	Arginine (M00844)	True
['True(K00611[score=413.8,thr=280.33,e=1.7e-124,strength=Strong])',
'True(K01940[score=629.8,thr=144.7,e=1.4999999999999998e-189,strength=Strong])',
'True(K01755[score=664.9,thr=175.9,e=2.9e-200,strength=Strong])']
ADCAI	Tryptophan (M00023)	False	['False',
'True(K01817[score=199.2,thr=207.3,e=2.2000000000000003e-59,strength=Possibly Strong]; K01609[score=284.8,thr=314.17,e=2.8e-85,strength=Possibly Strong])',
'True(K01696[score=745.2,thr=617.63,e=2.6e-224,strength=Strong];
K01695[score=302.9,thr=86.6,e=5.8e-91,strength=Strong])']
ADCAI	Leucine (M00432)	True
['True(K01649[score=486.4,thr=453.87,e=3.4e-146,strength=Strong])',
'True(K01704[score=240.4,thr=121.2,e=4.6e-72,strength=Strong];
K01703[score=579.5,thr=471.07,e=1.4e-174,strength=Strong])',
'True(K00052[score=501.3,thr=422.13,e=8.2e-151,strength=Strong])']
ADCAI	Lysine (M00016) True
['True(K00928[score=468.5,thr=353.77,e=5.7e-141,strength=Strong])',
'True(K00133[score=339.1,thr=189.07,e=1e-101,strength=Strong])',
'True(K01714[score=342.8,thr=257.53,e=5.4e-103,strength=Strong])',
'True(K00215[score=236.8,thr=131.67,e=8.4e-71,strength=Strong])',
'True(K00674[score=326.8,thr=153.07,e=5e-98,strength=Strong])',
'True(K00821[score=494.0,thr=473.83,e=8.700000000000001e-149,strength=Strong])',
'True(K01439[score=254.4,thr=276.5,e=4.4e-76,strength=Possibly Strong])',
'True(K01778[score=281.6,thr=70.8,e=1.6e-84,strength=Strong])',
'True(K01586[score=435.2,thr=272.33,e=7.8e-131,strength=Strong])']
ADCAI	Lipoic acid (M00881)	True
['True(K03801[score=135.6,thr=244.27,e=4.8e-40,strength=Missing])',
'True(K03644[score=403.2,thr=168.0,e=2.6999999999999998e-121,strength=Strong])']
AFCAI	Threonine (M00018)	True
['True(K00928[score=467.7,thr=353.77,e=9.799999999999999e-141,strength=Strong])',
'True(K00133[score=335.6,thr=189.07,e=1.2e-100,strength=Strong])',
'True(K00003[score=488.0,thr=273.33,e=9.5e-147,strength=Strong])',
'True(K02204[score=277.9,thr=133.1,e=2.7999999999999997e-83,strength=Strong])',
'True(K01733[score=323.1,thr=265.67,e=7.5e-97,strength=Strong])']
AFCAI	Arginine (M00844)	True
['True(K00611[score=411.1,thr=280.33,e=1.2e-123,strength=Strong])',
'True(K01940[score=635.8,thr=144.7,e=2.2999999999999998e-191,strength=Strong])',
'True(K01755[score=664.7,thr=175.9,e=3.5999999999999996e-200,strength=Strong])']
AFCAI	Tryptophan (M00023)	True
['True(K01657[score=572.6,thr=466.47,e=1.9999999999999998e-172,strength=Strong];
K01658[score=292.4,thr=241.3,e=4.1e-88,strength=Strong];

K00766[score=299.7,thr=94.77,e=6.6999999999999995e-90,strength=Strong])',
'True(K01817[score=200.1,thr=207.3,e=1.2e-59,strength=Possibly Strong];
K01609[score=283.6,thr=314.17,e=6.1e-85,strength=Possibly Strong])',
'True(K01696[score=747.0,thr=617.63,e=7.6e-225,strength=Strong];
K01695[score=302.6,thr=86.6,e=7.1e-91,strength=Strong])']
AFCAI Leucine (M00432) True
['True(K01649[score=487.3,thr=453.87,e=1.8000000000000001e-146,strength=Strong])',
'True(K01704[score=241.1,thr=121.2,e=2.8e-72,strength=Strong];
K01703[score=580.5,thr=471.07,e=6.8e-175,strength=Strong])',
'True(K00052[score=503.5,thr=422.13,e=1.9e-151,strength=Strong])']
AFCAI Lysine (M00016) True
['True(K00928[score=467.7,thr=353.77,e=9.799999999999999e-141,strength=Strong])',
'True(K00133[score=335.6,thr=189.07,e=1.2e-100,strength=Strong])',
'True(K01714[score=345.1,thr=257.53,e=1e-103,strength=Strong])',
'True(K00215[score=238.3,thr=131.67,e=2.8e-71,strength=Strong])',
'True(K00674[score=327.8,thr=153.07,e=2.6e-98,strength=Strong])',
'True(K00821[score=493.2,thr=473.83,e=1.5e-148,strength=Strong])',
'True(K01439[score=262.9,thr=276.5,e=1.1e-78,strength=Possibly Strong])',
'True(K01778[score=280.5,thr=70.8,e=3.3999999999999998e-84,strength=Strong])',
'True(K01586[score=433.5,thr=272.33,e=2.6e-130,strength=Strong])']
AFCAI Lipoic acid (M00881) False ['False',
'True(K03644[score=405.5,thr=168.0,e=5.5e-122,strength=Strong])']
BTB Threonine (M00018) True
['True(K00928[score=383.9,thr=353.77,e=2.5e-115,strength=Strong])',
'True(K00133[score=327.2,thr=189.07,e=4.2e-98,strength=Strong])',
'True(K00003[score=449.0,thr=273.33,e=5.9e-135,strength=Strong])',
'True(K02204[score=284.9,thr=133.1,e=2.2e-85,strength=Strong])',
'True(K01733[score=307.6,thr=265.67,e=3.6e-92,strength=Strong])']
BTB Arginine (M00844) False
['True(K00611[score=413.3,thr=280.33,e=2.5e-124,strength=Strong])',
'True(K01940[score=379.7,thr=144.7,e=7.2e-114,strength=Strong])', 'False']
BTB Tryptophan (M00023) True
['True(K01657[score=477.8,thr=466.47,e=1e-143,strength=Strong];
K01658[score=264.1,thr=241.3,e=1.7e-79,strength=Strong];
K00766[score=302.5,thr=94.77,e=9.499999999999999e-91,strength=Strong])',
'True(K01817[score=203.9,thr=207.3,e=8.4e-61,strength=Possibly Strong];
K01609[score=260.9,thr=314.17,e=4.9e-78,strength=Possibly Strong])',
'True(K01696[score=754.2,thr=617.63,e=5e-227,strength=Strong];
K01695[score=300.2,thr=86.6,e=3.7999999999999997e-90,strength=Strong])']
BTB Leucine (M00432) True
['True(K01649[score=483.0,thr=453.87,e=3.5e-145,strength=Strong])',
'True(K01704[score=237.5,thr=121.2,e=3.5000000000000003e-71,strength=Strong];
K01703[score=571.3,thr=471.07,e=4.1e-172,strength=Strong])',
'True(K00052[score=481.1,thr=422.13,e=1.1e-144,strength=Strong])']
BTB Lysine (M00016) False
['True(K00928[score=383.9,thr=353.77,e=2.5e-115,strength=Strong])',
'True(K00133[score=327.2,thr=189.07,e=4.2e-98,strength=Strong])',
'True(K01714[score=336.6,thr=257.53,e=4e-101,strength=Strong])', 'False',
'True(K00674[score=324.7,thr=153.07,e=2.2e-97,strength=Strong])',
'True(K00821[score=475.6,thr=473.83,e=3.2e-143,strength=Strong])',

```
'True(K01439[score=248.3,thr=276.5,e=3e-74,strength=Possibly Strong])', 'False',
'False']
BTB     Lipoic acid (M00881)    False   ['False',
'True(K03644[score=389.6,thr=168.0,e=3.6e-117,strength=Strong])']
BTQ     Threonine (M00018)      True
['True(K00928[score=385.8,thr=353.77,e=6.299999999999999e-116,strength=Strong])',
'True(K00133[score=327.7,thr=189.07,e=2.9e-98,strength=Strong])',
'True(K00003[score=448.3,thr=273.33,e=9.7e-135,strength=Strong])',
'True(K02204[score=285.0,thr=133.1,e=2e-85,strength=Strong])',
'True(K01733[score=307.6,thr=265.67,e=3.6e-92,strength=Strong])']
BTQ     Arginine (M00844)       False
['True(K00611[score=413.2,thr=280.33,e=2.7e-124,strength=Strong])',
'True(K01940[score=376.3,thr=144.7,e=7.5e-113,strength=Strong])', 'False']
BTQ     Tryptophan (M00023)     True
['True(K01657[score=477.3,thr=466.47,e=1.4e-143,strength=Strong];
K01658[score=264.0,thr=241.3,e=1.9e-79,strength=Strong];
K00766[score=302.5,thr=94.77,e=9.499999999999999e-91,strength=Strong])',
'True(K01817[score=203.9,thr=207.3,e=8.4e-61,strength=Possibly Strong];
K01609[score=262.7,thr=314.17,e=1.4e-78,strength=Possibly Strong])',
'True(K01696[score=752.7,thr=617.63,e=1.3999999999999999e-226,strength=Strong];
K01695[score=298.3,thr=86.6,e=1.4000000000000001e-89,strength=Strong])']
BTQ     Leucine (M00432)        True
['True(K01649[score=483.0,thr=453.87,e=3.5e-145,strength=Strong])',
'True(K01704[score=237.5,thr=121.2,e=3.5000000000000003e-71,strength=Strong];
K01703[score=572.8,thr=471.07,e=1.5e-172,strength=Strong])',
'True(K00052[score=481.1,thr=422.13,e=1.1e-144,strength=Strong])']
BTQ     Lysine (M00016) False
['True(K00928[score=385.8,thr=353.77,e=6.299999999999999e-116,strength=Strong])',
'True(K00133[score=327.7,thr=189.07,e=2.9e-98,strength=Strong])',
'True(K01714[score=336.6,thr=257.53,e=4e-101,strength=Strong])', 'False',
'True(K00674[score=324.7,thr=153.07,e=2.2e-97,strength=Strong])',
'True(K00821[score=475.6,thr=473.83,e=3.2e-143,strength=Strong])',
'True(K01439[score=248.4,thr=276.5,e=2.9000000000000004e-74,strength=Possibly
Strong])', 'False', 'False']
BTQ     Lipoic acid (M00881)    False   ['False',
'True(K03644[score=389.6,thr=168.0,e=3.6e-117,strength=Strong])']
BTQVLC  Threonine (M00018)      True
['True(K00928[score=385.8,thr=353.77,e=6.299999999999999e-116,strength=Strong])',
'True(K00133[score=327.7,thr=189.07,e=2.9e-98,strength=Strong])',
'True(K00003[score=448.3,thr=273.33,e=9.7e-135,strength=Strong])',
'True(K02204[score=285.0,thr=133.1,e=2e-85,strength=Strong])',
'True(K01733[score=307.6,thr=265.67,e=3.6e-92,strength=Strong])']
BTQVLC  Arginine (M00844)       False
['True(K00611[score=413.2,thr=280.33,e=2.7e-124,strength=Strong])',
'True(K01940[score=376.3,thr=144.7,e=7.5e-113,strength=Strong])', 'False']
BTQVLC  Tryptophan (M00023)     True
['True(K01657[score=477.3,thr=466.47,e=1.4e-143,strength=Strong];
K01658[score=264.0,thr=241.3,e=1.9e-79,strength=Strong];
K00766[score=302.5,thr=94.77,e=9.499999999999999e-91,strength=Strong])',
'True(K01817[score=203.9,thr=207.3,e=8.4e-61,strength=Possibly Strong];
```

K01609[score=262.7,thr=314.17,e=1.4e-78,strength=Possibly Strong])',
'True(K01696[score=752.7,thr=617.63,e=1.3999999999999999e-226,strength=Strong];
K01695[score=298.3,thr=86.6,e=1.4000000000000001e-89,strength=Strong])']
BTQVLC Leucine (M00432)   True
['True(K01649[score=483.0,thr=453.87,e=3.5e-145,strength=Strong])',
'True(K01704[score=237.5,thr=121.2,e=3.5000000000000003e-71,strength=Strong];
K01703[score=572.8,thr=471.07,e=1.5e-172,strength=Strong])',
'True(K00052[score=475.7,thr=422.13,e=4.9e-143,strength=Strong])']
BTQVLC Lysine (M00016) False
['True(K00928[score=385.8,thr=353.77,e=6.299999999999999e-116,strength=Strong])',
'True(K00133[score=327.7,thr=189.07,e=2.9e-98,strength=Strong])',
'True(K01714[score=336.6,thr=257.53,e=4e-101,strength=Strong])', 'False',
'True(K00674[score=324.7,thr=153.07,e=2.2e-97,strength=Strong])',
'True(K00821[score=475.6,thr=473.83,e=3.2e-143,strength=Strong])',
'True(K01439[score=248.4,thr=276.5,e=2.9000000000000004e-74,strength=Possibly
Strong])', 'False', 'False']
BTQVLC Lipoic acid (M00881)   False   ['False',
'True(K03644[score=389.6,thr=168.0,e=3.6e-117,strength=Strong])']
BTZ1   Threonine (M00018)   True
['True(K00928[score=382.6,thr=353.77,e=5.7e-115,strength=Strong])',
'True(K00133[score=326.8,thr=189.07,e=5.4000000000000004e-98,strength=Strong])',
'True(K00003[score=448.1,thr=273.33,e=1.1e-134,strength=Strong])',
'True(K02204[score=284.1,thr=133.1,e=3.7e-85,strength=Strong])',
'True(K01733[score=308.4,thr=265.67,e=2.1e-92,strength=Strong])']
BTZ1   Arginine (M00844)   False
['True(K00611[score=414.8,thr=280.33,e=9.1e-125,strength=Strong])',
'True(K01940[score=378.9,thr=144.7,e=1.1999999999999999e-113,strength=Strong])',
'False']
BTZ1   Tryptophan (M00023)   True
['True(K01657[score=477.3,thr=466.47,e=1.4e-143,strength=Strong];
K01658[score=266.5,thr=241.3,e=3.2000000000000003e-80,strength=Strong];
K00766[score=300.1,thr=94.77,e=4.9e-90,strength=Strong])',
'True(K01817[score=202.3,thr=207.3,e=2.5e-60,strength=Possibly Strong];
K01609[score=263.0,thr=314.17,e=1.1e-78,strength=Possibly Strong])',
'True(K01696[score=752.7,thr=617.63,e=1.3999999999999999e-226,strength=Strong];
K01695[score=301.1,thr=86.6,e=2e-90,strength=Strong])']
BTZ1   Leucine (M00432)   True
['True(K01649[score=484.2,thr=453.87,e=1.5000000000000001e-145,strength=Strong])',
'True(K01704[score=237.5,thr=121.2,e=3.7000000000000005e-71,strength=Strong];
K01703[score=573.8,thr=471.07,e=7.1e-173,strength=Strong])',
'True(K00052[score=481.1,thr=422.13,e=1.1e-144,strength=Strong])']
BTZ1   Lysine (M00016) False
['True(K00928[score=382.6,thr=353.77,e=5.7e-115,strength=Strong])',
'True(K00133[score=326.8,thr=189.07,e=5.4000000000000004e-98,strength=Strong])',
'True(K01714[score=336.6,thr=257.53,e=4e-101,strength=Strong])', 'False',
'True(K00674[score=324.4,thr=153.07,e=2.7e-97,strength=Strong])',
'True(K00821[score=478.0,thr=473.83,e=6e-144,strength=Strong])',
'True(K01439[score=247.8,thr=276.5,e=4.2e-74,strength=Possibly Strong])', 'False',
'False']
BTZ1   Lipoic acid (M00881)   False   ['False',

'True(K03644[score=389.6,thr=168.0,e=3.6e-117,strength=Strong])']
China	Threonine (M00018)	True	['True(K00928[score=302.3,thr=353.77,e=1.1999999999999999e-90,strength=Possibly Strong])', 'True(K00133[score=327.7,thr=189.07,e=2.9e-98,strength=Strong])', 'True(K00003[score=448.3,thr=273.33,e=9.7e-135,strength=Strong])', 'True(K02204[score=285.0,thr=133.1,e=2e-85,strength=Strong])', 'True(K01733[score=307.6,thr=265.67,e=3.6e-92,strength=Strong])']
China	Arginine (M00844)	False	['True(K00611[score=413.2,thr=280.33,e=2.7e-124,strength=Strong])', 'True(K01940[score=190.5,thr=144.7,e=1.3e-56,strength=Strong])', 'False']
China	Tryptophan (M00023)	True	['True(K01657[score=477.3,thr=466.47,e=1.4e-143,strength=Strong]; K01658[score=264.0,thr=241.3,e=1.9e-79,strength=Strong]; K00766[score=302.5,thr=94.77,e=9.499999999999999e-91,strength=Strong])', 'True(K01817[score=203.9,thr=207.3,e=8.4e-61,strength=Possibly Strong]; K01609[score=262.7,thr=314.17,e=1.4e-78,strength=Possibly Strong])', 'True(K01696[score=747.5,thr=617.63,e=5.1000000000000005e-225,strength=Strong]; K01695[score=298.3,thr=86.6,e=1.4000000000000001e-89,strength=Strong])']
China	Leucine (M00432)	False	['True(K01649[score=483.0,thr=453.87,e=3.5e-145,strength=Strong])', 'True(K01704[score=237.5,thr=121.2,e=3.5000000000000003e-71,strength=Strong]; K01703[score=572.8,thr=471.07,e=1.5e-172,strength=Strong])', 'False']
China	Lysine (M00016)	False	['True(K00928[score=302.3,thr=353.77,e=1.1999999999999999e-90,strength=Possibly Strong])', 'True(K00133[score=327.7,thr=189.07,e=2.9e-98,strength=Strong])', 'True(K01714[score=336.6,thr=257.53,e=4e-101,strength=Strong])', 'False', 'True(K00674[score=324.7,thr=153.07,e=2.2e-97,strength=Strong])', 'True(K00821[score=475.6,thr=473.83,e=3.2e-143,strength=Strong])', 'True(K01439[score=248.4,thr=276.5,e=2.9000000000000004e-74,strength=Possibly Strong])', 'False', 'False']
China	Lipoic acid (M00881)	False	['False', 'True(K03644[score=389.6,thr=168.0,e=3.6e-117,strength=Strong])']
China1	Threonine (M00018)	True	['True(K00928[score=382.6,thr=353.77,e=5.7e-115,strength=Strong])', 'True(K00133[score=327.7,thr=189.07,e=2.9e-98,strength=Strong])', 'True(K00003[score=448.1,thr=273.33,e=1.1e-134,strength=Strong])', 'True(K02204[score=282.1,thr=133.1,e=1.5e-84,strength=Strong])', 'True(K01733[score=308.4,thr=265.67,e=2.1e-92,strength=Strong])']
China1	Arginine (M00844)	False	['True(K00611[score=412.9,thr=280.33,e=3.3000000000000003e-124,strength=Strong])', 'True(K01940[score=373.8,thr=144.7,e=4.5e-112,strength=Strong])', 'False']
China1	Tryptophan (M00023)	True	['True(K01657[score=477.6,thr=466.47,e=1.1e-143,strength=Strong]; K01658[score=266.5,thr=241.3,e=3.2000000000000003e-80,strength=Strong]; K00766[score=300.2,thr=94.77,e=4.7e-90,strength=Strong])', 'True(K01817[score=202.3,thr=207.3,e=2.5e-60,strength=Possibly Strong]; K01609[score=263.0,thr=314.17,e=1.1e-78,strength=Possibly Strong])', 'True(K01696[score=752.6,thr=617.63,e=1.4999999999999998e-226,strength=Strong]; K01695[score=301.1,thr=86.6,e=2e-90,strength=Strong])']
China1	Leucine (M00432)	True

['True(K01649[score=483.6,thr=453.87,e=2.3e-145,strength=Strong])',
'True(K01704[score=237.9,thr=121.2,e=2.7e-71,strength=Strong];
K01703[score=574.0,thr=471.07,e=6.3999999999999999e-173,strength=Strong])',
'True(K00052[score=482.5,thr=422.13,e=4.3000000000000006e-145,strength=Strong])']
China1 Lysine (M00016) False
['True(K00928[score=382.6,thr=353.77,e=5.7e-115,strength=Strong])',
'True(K00133[score=327.7,thr=189.07,e=2.9e-98,strength=Strong])',
'True(K01714[score=336.6,thr=257.53,e=4e-101,strength=Strong])', 'False',
'True(K00674[score=324.4,thr=153.07,e=2.7e-97,strength=Strong])',
'True(K00821[score=476.3,thr=473.83,e=2e-143,strength=Strong])',
'True(K01439[score=247.8,thr=276.5,e=4.2e-74,strength=Possibly Strong])', 'False',
'False']
China1 Lipoic acid (M00881) False ['False',
'True(K03644[score=392.2,thr=168.0,e=5.8000000000000005e-118,strength=Strong])']
MEAM1 Threonine (M00018) True
['True(K00928[score=383.9,thr=353.77,e=2.5e-115,strength=Strong])',
'True(K00133[score=327.2,thr=189.07,e=4.2e-98,strength=Strong])',
'True(K00003[score=449.0,thr=273.33,e=5.9e-135,strength=Strong])',
'True(K02204[score=284.9,thr=133.1,e=2.2e-85,strength=Strong])',
'True(K01733[score=307.6,thr=265.67,e=3.6e-92,strength=Strong])']
MEAM1 Arginine (M00844) False
['True(K00611[score=413.3,thr=280.33,e=2.5e-124,strength=Strong])',
'True(K01940[score=379.7,thr=144.7,e=7.2e-114,strength=Strong])', 'False']
MEAM1 Tryptophan (M00023) True
['True(K01657[score=477.8,thr=466.47,e=1e-143,strength=Strong];
K01658[score=264.1,thr=241.3,e=1.7e-79,strength=Strong];
K00766[score=302.5,thr=94.77,e=9.499999999999999e-91,strength=Strong])',
'True(K01817[score=203.9,thr=207.3,e=8.4e-61,strength=Possibly Strong];
K01609[score=260.9,thr=314.17,e=4.9e-78,strength=Possibly Strong])',
'True(K01696[score=754.2,thr=617.63,e=5e-227,strength=Strong];
K01695[score=300.2,thr=86.6,e=3.7999999999999997e-90,strength=Strong])']
MEAM1 Leucine (M00432) True
['True(K01649[score=483.0,thr=453.87,e=3.5e-145,strength=Strong])',
'True(K01704[score=237.5,thr=121.2,e=3.5000000000000003e-71,strength=Strong];
K01703[score=571.3,thr=471.07,e=4.1e-172,strength=Strong])',
'True(K00052[score=481.1,thr=422.13,e=1.1e-144,strength=Strong])']
MEAM1 Lysine (M00016) False
['True(K00928[score=383.9,thr=353.77,e=2.5e-115,strength=Strong])',
'True(K00133[score=327.2,thr=189.07,e=4.2e-98,strength=Strong])',
'True(K01714[score=336.6,thr=257.53,e=4e-101,strength=Strong])', 'False',
'True(K00674[score=324.7,thr=153.07,e=2.2e-97,strength=Strong])',
'True(K00821[score=475.6,thr=473.83,e=3.2e-143,strength=Strong])',
'True(K01439[score=248.3,thr=276.5,e=3e-74,strength=Possibly Strong])', 'False',
'False']
MEAM1 Lipoic acid (M00881) False ['False',
'True(K03644[score=389.6,thr=168.0,e=3.6e-117,strength=Strong])']
PeMo Threonine (M00018) True
['True(K00928[score=451.4,thr=353.77,e=8.5e-136,strength=Strong])',
'True(K00133[score=335.6,thr=189.07,e=1.2e-100,strength=Strong])',
'True(K00003[score=479.3,thr=273.33,e=4e-144,strength=Strong])',

```
'True(K02204[score=294.0,thr=133.1,e=3.9e-88,strength=Strong])',
'True(K01733[score=319.9,thr=265.67,e=7.0999999999999995e-96,strength=Strong])']
PeMo    Arginine (M00844)       True
['True(K00611[score=418.8,thr=280.33,e=5.6000000000000005e-126,strength=Strong])',
'True(K01940[score=650.4,thr=144.7,e=9e-196,strength=Strong])',
'True(K01755[score=661.8,thr=175.9,e=2.6e-199,strength=Strong])']
PeMo    Tryptophan (M00023)     True
['True(K01657[score=540.6,thr=466.47,e=9.8e-163,strength=Strong];
K01658[score=277.6,thr=241.3,e=1.2999999999999999e-83,strength=Strong];
K00766[score=295.5,thr=94.77,e=1.2e-88,strength=Strong])',
'True(K01817[score=207.1,thr=207.3,e=9.2e-62,strength=Possibly Strong];
K01609[score=286.9,thr=314.17,e=6.2000000000000005e-86,strength=Possibly Strong])',
'True(K01696[score=749.3,thr=617.63,e=1.5e-225,strength=Strong];
K01695[score=306.9,thr=86.6,e=3.5e-92,strength=Strong])']
PeMo    Leucine (M00432)        True
['True(K01649[score=483.8,thr=453.87,e=2.0000000000000002e-145,strength=Strong])',
'True(K01704[score=236.8,thr=121.2,e=5.9999999999999995e-71,strength=Strong];
K01703[score=585.5,thr=471.07,e=2.1e-176,strength=Strong])',
'True(K00052[score=509.5,thr=422.13,e=2.8e-153,strength=Strong])']
PeMo    Lysine (M00016) True
['True(K00928[score=451.4,thr=353.77,e=8.5e-136,strength=Strong])',
'True(K00133[score=335.6,thr=189.07,e=1.2e-100,strength=Strong])',
'True(K01714[score=333.8,thr=257.53,e=2.9000000000000003e-100,strength=Strong])',
'True(K00215[score=243.7,thr=131.67,e=6.4e-73,strength=Strong])',
'True(K00674[score=322.9,thr=153.07,e=7.8e-97,strength=Strong])',
'True(K00821[score=493.0,thr=473.83,e=1.6999999999999998e-148,strength=Strong])',
'True(K01439[score=252.3,thr=276.5,e=1.9e-75,strength=Possibly Strong])',
'True(K01778[score=261.3,thr=70.8,e=2.4e-78,strength=Strong])',
'True(K01586[score=451.3,thr=272.33,e=1e-135,strength=Strong])']
PeMo    Lipoic acid (M00881)    False   ['False',
'True(K03644[score=387.1,thr=168.0,e=2.1e-116,strength=Strong])']
SiSi    Threonine (M00018)      True
['True(K00928[score=413.9,thr=353.77,e=2.0000000000000003e-124,strength=Strong])',
'True(K00133[score=339.6,thr=189.07,e=7.4e-102,strength=Strong])',
'True(K00003[score=452.7,thr=273.33,e=4.5e-136,strength=Strong])',
'True(K02204[score=140.4,thr=133.1,e=1.5e-41,strength=Strong])',
'True(K01733[score=313.8,thr=265.67,e=4.7e-94,strength=Strong])']
SiSi    Arginine (M00844)       True
['True(K00611[score=407.0,thr=280.33,e=2.1e-122,strength=Strong])',
'True(K01940[score=543.8,thr=144.7,e=1.6e-163,strength=Strong])',
'True(K01755[score=443.8,thr=175.9,e=2.5000000000000004e-133,strength=Strong])']
SiSi    Tryptophan (M00023)     False
['True(K01657[score=479.1,thr=466.47,e=4e-144,strength=Strong];
K01658[score=275.9,thr=241.3,e=4.5e-83,strength=Strong];
K00766[score=314.5,thr=94.77,e=2.2e-94,strength=Strong])', 'False',
'True(K01696[score=750.3,thr=617.63,e=7.699999999999999e-226,strength=Strong];
K01695[score=288.9,thr=86.6,e=9.999999999999999e-87,strength=Strong])']
SiSi    Leucine (M00432)        True
['True(K01649[score=475.2,thr=453.87,e=8.3e-143,strength=Strong])',
'True(K01704[score=239.2,thr=121.2,e=1.1e-71,strength=Strong];
```

K01703[score=589.2,thr=471.07,e=1.4999999999999998e-177,strength=Strong])',
'True(K00052[score=496.0,thr=422.13,e=3.5e-149,strength=Strong])']
SiSi   Lysine (M00016) False
['True(K00928[score=413.9,thr=353.77,e=2.0000000000000003e-124,strength=Strong])',
'True(K00133[score=339.6,thr=189.07,e=7.4e-102,strength=Strong])',
'True(K01714[score=337.5,thr=257.53,e=2.1e-101,strength=Strong])',
'True(K00215[score=251.9,thr=131.67,e=2.0999999999999999e-75,strength=Strong])',
'True(K00674[score=314.7,thr=153.07,e=2.3e-94,strength=Strong])',
'True(K00821[score=489.7,thr=473.83,e=1.7999999999999998e-147,strength=Strong])',
'True(K01439[score=251.5,thr=276.5,e=3.3e-75,strength=Possibly Strong])', 'False',
'False']
SiSi   Lipoic acid (M00881)   False   ['False',
'True(K03644[score=399.1,thr=168.0,e=4.7e-120,strength=Strong])']
TV   Threonine (M00018)   True
['True(K00928[score=425.4,thr=353.77,e=6.6e-128,strength=Strong])',
'True(K00133[score=329.0,thr=189.07,e=1.2e-98,strength=Strong])',
'True(K00003[score=457.1,thr=273.33,e=2.1e-137,strength=Strong])',
'True(K02204[score=295.1,thr=133.1,e=1.7e-88,strength=Strong])',
'True(K01733[score=318.0,thr=265.67,e=2.5e-95,strength=Strong])']
TV   Arginine (M00844)   True
['True(K00611[score=418.2,thr=280.33,e=8.400000000000001e-126,strength=Strong])',
'True(K01940[score=651.1,thr=144.7,e=5.400000000000001e-196,strength=Strong])',
'True(K01755[score=667.2,thr=175.9,e=6.2000000000000005e-201,strength=Strong])']
TV   Tryptophan (M00023)   True
['True(K01657[score=567.0,thr=466.47,e=9.9e-171,strength=Strong];
K01658[score=285.4,thr=241.3,e=5.5e-86,strength=Strong];
K00766[score=295.0,thr=94.77,e=1.8e-88,strength=Strong])',
'True(K01817[score=200.4,thr=207.3,e=9.500000000000001e-60,strength=Possibly
Strong]; K01609[score=282.6,thr=314.17,e=1.2e-84,strength=Possibly Strong])',
'True(K01696[score=741.2,thr=617.63,e=4.2000000000000004e-223,strength=Strong];
K01695[score=305.9,thr=86.6,e=7.1e-92,strength=Strong])']
TV   Leucine (M00432)   True
['True(K01649[score=485.3,thr=453.87,e=7.3000000000000005e-146,strength=Strong])',
'True(K01704[score=235.4,thr=121.2,e=1.5e-70,strength=Strong];
K01703[score=588.3,thr=471.07,e=3e-177,strength=Strong])',
'True(K00052[score=503.2,thr=422.13,e=2.3e-151,strength=Strong])']
TV   Lysine (M00016) True
['True(K00928[score=425.4,thr=353.77,e=6.6e-128,strength=Strong])',
'True(K00133[score=329.0,thr=189.07,e=1.2e-98,strength=Strong])',
'True(K01714[score=346.2,thr=257.53,e=5.2e-104,strength=Strong])',
'True(K00215[score=244.2,thr=131.67,e=4.8e-73,strength=Strong])',
'True(K00674[score=316.1,thr=153.07,e=8.9e-95,strength=Strong])',
'True(K00821[score=500.1,thr=473.83,e=1.1999999999999999e-150,strength=Strong])',
'True(K01439[score=253.1,thr=276.5,e=1.0999999999999999e-75,strength=Possibly
Strong])', 'True(K01778[score=257.3,thr=70.8,e=3.9e-77,strength=Strong])',
'True(K01586[score=455.4,thr=272.33,e=6.1e-137,strength=Strong])']
TV   Lipoic acid (M00881)   True
['True(K03801[score=135.4,thr=244.27,e=5.3e-40,strength=Missing])',
'True(K03644[score=392.3,thr=168.0,e=5.600000000000001e-118,strength=Strong])']
ARAD   Lipoic acid biosynthesis, plants and bacteria, octanoyl-ACP =>

dihydrolipoyl-E2/H (2) (complete 2/2) (M00881) True
['True(K03644[score=466.9,thr=168.0,e=2.9e-140,strength=Strong])',
'True(K03801[score=260.0,thr=244.27,e=1.4e-77,strength=Strong])']
ARAF   Biotin biosynthesis, pimeloyl-ACP/CoA => biotin (4) (complete 3/3)
(M00123)   True
['True(K00652[score=473.7,thr=459.97,e=3.3999999999999996e-142,strength=Strong])',
'True(K00833[score=685.0,thr=420.7,e=1.7e-206,strength=Strong])',
'True(K01012[score=518.1,thr=161.93,e=1.1e-155,strength=Strong])',
'True(K01935[score=237.1,thr=122.5,e=1.3e-70,strength=Strong])']
ARAF   Pyridoxal phosphate biosynthesis, erythrose-4P => pyridoxal-P (6)
(complete 6/6) (M00124) True
['True(K00097[score=495.4,thr=430.27,e=1.0999999999999999e-148,strength=Strong])',
'True(K00275[score=268.1,thr=32.63,e=6.600000000000001e-80,strength=Strong])',
'True(K00831[score=538.7,thr=131.1,e=4.5e-162,strength=Strong])',
'True(K03472[score=572.4,thr=483.47,e=1.1e-172,strength=Strong])',
'True(K03473[score=540.3,thr=276.97,e=1.2e-162,strength=Strong])',
'True(K03474[score=412.5,thr=90.83,e=6.6000000000000005e-124,strength=Strong])']
ARAF   Tetrahydrofolate biosynthesis, GTP => THF (7) (complete 5/5) (M00126)
True   ['True(K00287[score=201.9,thr=61.57,e=6.9e-60,strength=Strong])',
'True(K00796[score=387.6,thr=235.83,e=2e-116,strength=Strong])',
'True(K00950[score=213.8,thr=42.53,e=1.3e-63,strength=Strong])',
'True(K01495[score=217.5,thr=55.27,e=8.8e-65,strength=Strong])',
'True(K01633[score=125.5,thr=93.17,e=1e-36,strength=Strong])',
'True(K08310[score=190.0,thr=96.27,e=1.2e-56,strength=Strong])',
'True(K11754[score=470.1,thr=373.63,e=7.1e-141,strength=Strong])']
ARAF   Thiamine biosynthesis, prokaryotes, AIR (+ DXP/tyrosine) => TMP/TPP (9)
(complete 7/7) (M00127) True
['True(K00788[score=203.2,thr=167.63,e=3e-60,strength=Strong])',
'True(K00941[score=369.5,thr=323.27,e=1.2e-110,strength=Strong])',
'True(K00946[score=400.9,thr=145.87,e=3.4e-120,strength=Strong])',
'True(K03147[score=1141.4,thr=578.6,e=0.0,strength=Strong])',
'True(K03148[score=334.6,thr=331.2,e=2.3e-100,strength=Strong])',
'True(K03149[score=411.4,thr=166.5,e=2.5e-123,strength=Strong])',
'True(K03150[score=511.0,thr=168.77,e=1.1e-153,strength=Strong])',
'True(K03151[score=543.8,thr=119.0,e=2.2e-163,strength=Strong])',
'True(K03154[score=57.1,thr=34.8,e=5.6e-16,strength=Strong])']
ARAF   Lipoic acid biosynthesis, plants and bacteria, octanoyl-ACP =>
dihydrolipoyl-E2/H (2) (complete 2/2) (M00881) True
['True(K03644[score=474.0,thr=168.0,e=1.9999999999999997e-142,strength=Strong])',
'True(K03801[score=267.6,thr=244.27,e=6.900000000000001e-80,strength=Strong])']
CBTQ   Lipoic acid biosynthesis, plants and bacteria, octanoyl-ACP =>
dihydrolipoyl-E2/H (2) (complete 2/2) (M00881) True
['True(K03644[score=445.8,thr=168.0,e=4.5e-134,strength=Strong])',
'True(K03801[score=267.8,thr=244.27,e=3.8000000000000004e-80,strength=Strong])']
CBTQ   Pyridoxal phosphate biosynthesis, R5P + glyceraldehyde-3P + glutamine =>
pyridoxal-P (2) (complete 1/1) (M00916)   True
['True(K06215[score=497.3,thr=129.43,e=5.6e-150,strength=Strong])',
'True(K08681[score=131.4,thr=81.9,e=7.5e-39,strength=Strong])']
MEAM   NAD biosynthesis, aspartate => quinolinate => NAD (5) (complete 5/5)
(M00115)   True

['True(K00278[score=747.9,thr=544.6,e=3.9e-225,strength=Strong])',
'True(K00767[score=356.3,thr=215.2,e=4.3999999999999996e-107,strength=Strong])',
'True(K00969[score=223.3,thr=120.0,e=1.1e-66,strength=Strong])',
'True(K01950[score=457.8,thr=485.07,e=1.6999999999999998e-137,strength=Possibly
Strong])', 'True(K03517[score=445.8,thr=95.83,e=3.9e-134,strength=Strong])']
MEAM   Ubiquinone biosynthesis, prokaryotes, chorismate (+ polyprenyl-PP) =>
ubiquinol (9) (complete 8/8) (M00117) True
['True(K00568[score=357.8,thr=321.43,e=1.8e-107,strength=Strong])',
'True(K03179[score=401.6,thr=228.7,e=7.8e-121,strength=Strong])',
'True(K03181[score=169.8,thr=55.13,e=1.6e-50,strength=Strong])',
'True(K03182[score=825.3,thr=508.0,e=7.1e-249,strength=Strong])',
'True(K03183[score=390.4,thr=281.53,e=2.5e-117,strength=Strong])',
'True(K03184[score=571.7,thr=478.8,e=2.3e-172,strength=Strong])',
'True(K03185[score=461.0,thr=420.5,e=9.999999999999999e-139,strength=Strong])',
'True(K03186[score=295.3,thr=90.2,e=8.900000000000001e-89,strength=Strong])',
'True(K18800[score=568.1,thr=473.43,e=6.299999999999999e-171,strength=Strong])']
MEAM   Coenzyme A biosynthesis, pantothenate => CoA (4) (complete 3/3) (M00120)
True   ['True(K00859[score=228.4,thr=54.77,e=3e-68,strength=Strong])',
'True(K00867[score=512.0,thr=135.97,e=2.2e-154,strength=Strong])',
'True(K00954[score=246.7,thr=68.73,e=6e-74,strength=Strong])',
'True(K13038[score=582.8,thr=246.9,e=1.2e-175,strength=Strong])']
MEAM   Pimeloyl-ACP biosynthesis, BioC-BioH pathway, malonyl-ACP => pimeloyl-ACP
(6) (complete 6/6) (M00572)   True
['True(K00059[score=317.9,thr=264.9,e=2e-95,strength=Strong])',
'True(K00208[score=400.2,thr=269.37,e=2.7e-120,strength=Strong])',
'True(K00647[score=374.6,thr=369.0,e=3e-112,strength=Strong])',
'True(K02169[score=276.2,thr=166.77,e=1.0000000000000001e-82,strength=Strong])',
'True(K02170[score=266.8,thr=147.4,e=9e-80,strength=Strong])',
'True(K02372[score=218.4,thr=83.87,e=3.5e-65,strength=Strong])']
MEAM   Lipoic acid biosynthesis, plants and bacteria, octanoyl-ACP =>
dihydrolipoyl-E2/H (2) (complete 2/2) (M00881) True
['True(K03644[score=469.9,thr=168.0,e=2.3e-141,strength=Strong])',
'True(K03801[score=264.6,thr=244.27,e=3.7e-79,strength=Strong])']
MEAM   PreQ1 biosynthesis, GTP => 7-Aminomethyl-7-deazaguanine (5) (complete 5/5)
(M00988)   True
['True(K01495[score=196.1,thr=55.27,e=1.7000000000000002e-58,strength=Strong])',
'True(K01737[score=156.7,thr=26.63,e=1.8e-46,strength=Strong])',
'True(K06879[score=434.1,thr=216.7,e=1.6000000000000002e-130,strength=Strong])',
'True(K06920[score=304.2,thr=95.43,e=3.8e-91,strength=Strong])',
'True(K10026[score=207.0,thr=86.0,e=8.2e-62,strength=Strong])']
MEDQ2   Coenzyme A biosynthesis, pantothenate => CoA (4) (complete 3/3) (M00120)
True   ['True(K00859[score=236.5,thr=54.77,e=1.7e-70,strength=Strong])',
'True(K00867[score=506.2,thr=135.97,e=1.9999999999999997e-152,strength=Strong])',
'True(K00954[score=235.6,thr=68.73,e=2.3e-70,strength=Strong])',
'True(K13038[score=577.7,thr=246.9,e=6.4e-174,strength=Strong])']
MEDQ2   Riboflavin biosynthesis, plants and bacteria, GTP => riboflavin/FMN/FAD (7)
(complete 7/7) (M00125)   False
['True(K00793[score=243.0,thr=56.0,e=1.8e-72,strength=Strong])',
'True(K00794[score=214.0,thr=45.23,e=7.2e-64,strength=Strong])',
'True(K01497[score=308.5,thr=153.5,e=2.6e-92,strength=Strong])',

```
'True(K02858[score=361.7,thr=355.17,e=1.9e-108,strength=Strong])',
'True(K11752[score=492.3,thr=179.9,e=1.2e-147,strength=Strong])',
'True(K11753[score=417.7,thr=164.5,e=2.5000000000000004e-125,strength=Strong])',
'False']
MEDQ2   Lipoic acid biosynthesis, plants and bacteria, octanoyl-ACP =>
dihydrolipoyl-E2/H (2)  (complete 2/2) (M00881) True
['True(K03644[score=477.9,thr=168.0,e=1.3e-143,strength=Strong])',
'True(K03801[score=267.6,thr=244.27,e=7.100000000000001e-80,strength=Strong])']
RMEAM1  Lipoic acid biosynthesis, plants and bacteria, octanoyl-ACP =>
dihydrolipoyl-E2/H (2)  (complete 2/2) (M00881) True
['True(K03644[score=486.5,thr=168.0,e=2.1e-146,strength=Strong])',
'True(K03801[score=235.6,thr=244.27,e=2.3e-70,strength=Possibly Strong])']
RMEAM1  PreQ1 biosynthesis, GTP => 7-Aminomethyl-7-deazaguanine (5)  (complete 5/5)
(M00988)        True
['True(K01495[score=255.5,thr=55.27,e=1.5e-76,strength=Strong])',
'True(K01737[score=103.5,thr=26.63,e=3.3e-30,strength=Strong])',
'True(K06879[score=405.4,thr=216.7,e=7.999999999999999e-122,strength=Strong])',
'True(K06920[score=310.2,thr=95.43,e=5.3e-93,strength=Strong])',
'True(K10026[score=171.5,thr=86.0,e=5.1e-51,strength=Strong])']
wChina1 Lipoic acid biosynthesis, plants and bacteria, octanoyl-ACP =>
dihydrolipoyl-E2/H (2)  (complete 2/2) (M00881) True
['True(K03644[score=457.4,thr=168.0,e=2.1e-137,strength=Strong])',
'True(K03801[score=235.1,thr=244.27,e=5.3e-70,strength=Possibly Strong])']
wbt     Lipoic acid biosynthesis, plants and bacteria, octanoyl-ACP =>
dihydrolipoyl-E2/H (2)  (complete 2/2) (M00881) True
['True(K03644[score=478.5,thr=168.0,e=8.4e-144,strength=Strong])',
'True(K03801[score=233.2,thr=244.27,e=2e-69,strength=Possibly Strong])']
```
