# Supplementary material for: Comparative genomics and metabolic model analysis of primary and secondary endosymbionts present in different species of whitefly
Source: J Insect Sci. 2026 Aug 8;26(4):ieag083. doi: 10.1093/jisesa/ieag083 (PMC13452057; doi:10.1093/jisesa/ieag083)
Supplement: Supplementary file 1 [file ieag083_supplementary_data.zip › Supplementary Table S8.docx]

1) Code for analysis of mutational pressure: Zenodo DOI: https://doi.org/10.5281/zenodo.21283855
2) Arginine pathway specific GEM script — Zenodo DOI: https://doi.org/10.5281/zenodo.21283855
3) Threonine pathway specific GEM script — Zenodo DOI: https://doi.org/10.5281/zenodo.21283855
4) Tryptophan pathway specific GEM script — Zenodo DOI: https://doi.org/10.5281/zenodo.21283855
5) Lysine pathway specific GEM script — Zenodo DOI: https://doi.org/10.5281/zenodo.21283855
6) Leucine pathway specific GEM script — Zenodo DOI: https://doi.org/10.5281/zenodo.21283855
7) Phenylalanine pathway specific GEM script — Zenodo DOI: https://doi.org/10.5281/zenodo.21283855
