# Supplementary material for: Comparative genomics and metabolic model analysis of primary and secondary endosymbionts present in different species of whitefly
Source: J Insect Sci. 2026 Aug 8;26(4):ieag083. doi: 10.1093/jisesa/ieag083 (PMC13452057; doi:10.1093/jisesa/ieag083)
Supplement: Supplementary file 1 [file ieag083_supplementary_data.zip › Supplementary Table S9.docx]

**Supplementary Table S9**: Table depicts exchange bounds for metabolites in the phenylalanine biosynthesis model under minimal and host‑supplemented media.

| **Metabolite** | **KEGG ID** | **Minimal medium exchange bounds** | **Host‑supplemented exchange bounds** |
| --- | --- | --- | --- |
| **Chorismate** | C00251 | –1000 to 1000 | –1000 to 1000 |
| **Prephenate** | C00254 | Not allowed | –1000 to 1000 |
| **Phenylpyruvate** | C00166 | Not allowed | –1000 to 1000 |
| **L‑Phenylalanine** | C00079 | Demand reaction only (DM_C00079) | Demand reaction only (DM_C00079) |
| **L‑Glutamate** | C00025 | –1000 to 1000 | –1000 to 1000 |
| **2‑Oxoglutarate** | C00026 | –1000 to 1000 | –1000 to 1000 |
| **H₂O** | C00001 | –1000 to 1000 | –1000 to 1000 |
| **CO₂** | C00011 | –1000 to 1000 | –1000 to 1000 |
